# BMP-7 mRNA delivered by Fibrin–CaP scaffolds activates osteogenic programs in vivo as evidenced by transcriptomic and proteomic analyses^☆^^☆☆^

**DOI:** 10.1016/j.bioactmat.2026.05.046

**Published:** 2026-05-29

**Authors:** Claudia Del Toro Runzer, Nadia Roumans, Freek G. Bouwman, Betzabeth Pereira Herrera, Berta Cillero-Pastor, Micaela Roque, Joëlle Amédée, Elise Bovine, Florence Barrère de Groot, Christian Plank, Andrea Banfi, Nunzia di Maggio, Martijn van Griensven, Elizabeth R. Balmayor

**Affiliations:** a Department of Cell Biology-Inspired Tissue Engineering, MERLN Institute for Technology-Inspired Regenerative Medicine, Maastricht University, Maastricht, the Netherlands; b Department of Human Biology, Maastricht University, Maastricht, the Netherlands; c Tissue Bioengineering Laboratory (BioTis), Inserm U1026, University of Bordeaux, Bordeaux, France; d OZ Biosciences SAS, Marseille, France; e Kuros Biosciences BV, Bilthoven, the Netherlands; f Ethris GmbH, Planegg, Germany; g Cell and Gene Therapy, Department of Biomedicine, Basel University Hospital and University of Basel, Basel, Switzerland; h Musculoskeletal Gene Therapy Laboratory, Rehabilitation Medicine Research Center, Mayo Clinic, Rochester, MN, USA; i Experimental Orthopaedics and Trauma Surgery, RWTH Aachen University Hospital, Aachen, Germany; j Department of Orthopaedic, Trauma, and Reconstructive Surgery, RWTH Aachen University Hospital, Aachen, Germany

**Keywords:** Bone morphogenetic protein 7, Chemically modified mRNA, Lipids, Osteogenesis, Ossification, Ectopic bone formation, Bone healing

## Abstract

Two recombinant bone morphogenetic proteins (BMP-2 and BMP-7) have received FDA approval for bone-related therapies. However, their clinical performance is limited by high costs, the need for supraphysiological doses, and adverse side effects. Here, we describe a chemically modified mRNA (cmRNA) encoding BMP-7 that promotes osteogenesis and functional ossification. The BMP-7 cmRNA is delivered using optimized lipid vectors and a composite fibrin–calcium phosphate scaffold. Among several lipids evaluated, two previously unexplored lipids efficiently condense mRNA and mediate its *in vivo* delivery. Transfer of BMP-7 cmRNA lipoplexes to human mesenchymal stromal cells activates intracellular vesicle transport and cytoskeletal remodeling, and regulates extracellular matrix production and calcium-associated processes. These responses were accompanied by robust mineralization and activation of key osteogenic pathways. *In vivo*, BMP-7 mRNA-activated scaffolds promote the formation of ossified tissue, with the highest dose yielding the largest ectopic bony growth. We further observe concurrent angiogenesis and neurogenesis, demonstrating coordinated tissue regeneration. This platform enables effective *in vivo* mRNA delivery for bone healing and can be applied to other tissues, facilitating the development of mRNA therapeutics in regenerative medicine.

## Highlights

• Osteogenesis and functional ossification induced *in vitro* and *in vivo* by a BMP-7 encoding chemically modified mRNA (cmRNA).
• Lipid vectors enable efficient BMP-7 cmRNA delivery to human mesenchymal stromal cells, driving robust BMP-7 production and mineralization.
• BMP-7 cmRNA-activated fibrin–calcium phosphate matrix supported local cmRNA retention and dose-dependent ossified tissue formation *in vivo.*
• Proteomics and transcriptomics identify distinct mRNA-dependent mechanisms and pathways underpinning BMP-7–mediated osteogenesis.
• BMP-7 cmRNA transcript-activated matrix promoted tissue regeneration, with evidence of angiogenesis, innervation, and ossification *in vivo.*

## Graphical abstract

**Figure:**
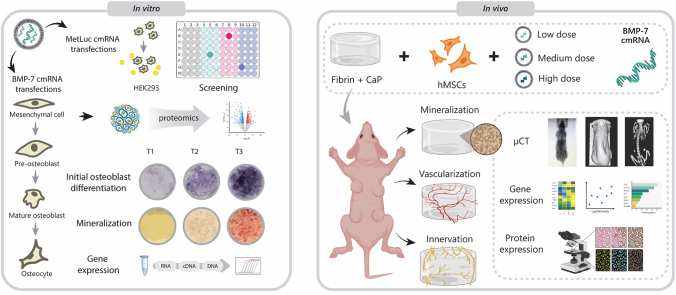

## 1 Introduction

Bone Morphogenetic Protein-7 (BMP-7), also known as human osteogenic protein-1 (OP-1®), is a crucial mediator of fracture healing. It stimulates the migration, proliferation, and osteogenic differentiation of mesenchymal cells into bone-forming osteoblasts [1]. The efficacy of recombinant human BMP-7 (rhBMP-7) in promoting bone regeneration has been extensively validated in preclinical studies [2], clinical trials [3,4], and clinical applications following its FDA approval in 2001 [5], specifically for spine fusions, open tibial fractures treated with intramedullary fixation, and tibial nonunions [6]. Its therapeutic scope has further expanded through off-label applications [[7], [8], [9]].

Clinically, rhBMP-7 is typically administered at a dose of 3.5 mg with 1 g of bovine collagen granules as a scaffold [10]. Although successful healing outcomes have been reported [11], the widespread adoption of rhBMP-7 has been hindered by high manufacturing costs [12] and dose-dependent adverse side effects. Reported complications include vertebral osteolysis, ectopic bone formation, and soft tissue swelling [9,13,14]. These complications largely stem from the supraphysiological doses required to compensate for rhBMP-7's short half-life. Ultimately, OP-1® was withdrawn from the market and restrictions were imposed on the clinical use of BMP-based therapies [15], underscoring a critical need for safer and more cost-effective alternatives.

We hypothesized that local delivery of chemically modified messenger RNA (cmRNA) encoding BMP-7 could effectively promote bone regeneration. We selected cmRNA over protein- and DNA-based therapeutics, and BMP-7 over the previously explored BMP-2 [[16], [17], [18]], for several reasons. First, protein expression following mRNA delivery need not be prolonged or excessive, as transgenic proteins elicit potent autocrine and paracrine effects in a protected pericellular environment [19], thereby enhancing signaling efficiency. Second, mRNA is delivered directly into the cytoplasm and efficiently translated, whereas DNA requires nuclear entry; each mRNA molecule can generate multiple protein copies. Third, protein-based BMP therapies have demonstrated limited clinical efficacy, and DNA-based strategies remain constrained by the potential risk of genomic integration. In contrast, mRNA-based therapeutics have shown an excellent safety profile, as evidenced by their widespread use in millions of individuals during the COVID-19 pandemic [20]. Fourth, cmRNA production is cost-effective, scalable [21,22], and performed in a cell-free *in vitro* process, ensuring consistent quality and eliminating contamination risks [23,24]. Finally, while BMP-2 cmRNA has been shown to promote bone healing [16,17], BMP-7 cmRNA represents a promising alternative based on literature suggesting distinct biological functions, including enhanced chondrogenic differentiation of mesenchymal stem cells [25], a process essential for endochondral ossification in long bones [26], and a comparatively lower inflammatory profile [27]. These characteristics may be particularly relevant for applications involving recalcitrant fractures.

Two critical aspects for the successful implementation of cmRNA technology in bone healing are efficient delivery of cmRNA into target cells and its localized administration to bone tissue. Intracellularly, lipid-mediated mRNA transfer has proven to be particularly effective in mRNA vaccination [28]. However, lipid formulations must be optimized for the specific target cell type and desired functionality [29,30]. Furthermore, for tissue-level administration, particularly in bone repair, a tridimensional scaffold is often required to bridge large defects and to retain therapeutic molecules at the site. Research to date has primally focused on proteins and DNA therapeutics; however, the need for a carrier specifically tailored for mRNA delivery, capable of amplifying its therapeutic benefits, remains unmet.

While mRNA-based delivery of osteogenic factors, particularly BMP-2, has been increasingly explored, the application of BMP-7 cmRNA remains largely underdeveloped despite its distinct biological role in bone formation. In addition, previous studies have generally focused on proof-of-concept delivery without systematically addressing vector optimization or downstream mechanistic responses. In this context, the present study combines *(i)* the use of BMP-7 cmRNA as an underexplored osteogenic trigger, *(ii)* a systematic, bottom-up screening strategy for lipid-based delivery vectors, and *(iii)* a composite fibrin-calcium phosphate scaffold designed to support localized and sustained mRNA activity. Furthermore, we integrate transcriptomic and proteomic analyses to elucidate the molecular pathways underlying the observed regenerative effects, providing a level of mechanistic insight that remains limited in current mRNA-based bone tissue engineering approaches.

Building on these considerations, we first evaluated various lipids as delivery vectors for transferring cmRNA into cells using reporter HEK cells and primary human mesenchymal stromal cells (hMSCs). The investigated lipids included two experimental formulations not previously reported for BMP cmRNA delivery. We assessed lipid performance based on protein production and cytotoxicity and identified optimal cmRNA-lipid nanoparticles. We then delivered the optimized BMP-7 cmRNA lipoplexes into hMSCs and assessed their osteogenic potential through functional assays and proteomics, which enabled the identification of early activated pathways. For effective *in vivo* delivery and sustained cmRNA activity, we developed a transcript-activated matrix (TAM) using a medical-grade fibrin scaffold supplemented with calcium phosphate (CaP) particles. In addition to fibrin's excellent tissue integration, low immunogenicity, and biodegradability [31,32], it has shown strong potential as a carrier for RNA delivery [33,34]. Importantly, fibrin also promotes angiogenesis [35] and supports neurogenesis in permissive environments [36,37], both of which are essential for bone healing. Combined with osteoinductive CaP particles, fibrin provides a suitable platform for BMP-7 cmRNA delivery, ensuring precisely regulated spatial and temporal activation of osteogenesis while simultaneously supporting angiogenesis and neurogenesis for comprehensive tissue regeneration. We validated this approach in a subcutaneous ectopic bone formation model in immunodeficient mice. The controlled environment of this model allowed us to attribute bone formation directly to the implanted cmRNA TAM, as it minimizes confounding factors such as natural bone healing stimuli [38]. This study aims to lay the groundwork for a cost-effective, scalable, and safer approach to bone regeneration therapy, offering a promising alternative to existing BMP-based treatments.

## 2 Methods

### 2.1 Chemically modified mRNA synthesis and formulation into lipoplexes

cmRNAs used in this study included those encoding *Metridia Luciferase* (MetLuc) and BMP-7. cmRNAs were produced by *in vitro* transcription (IVT), including chemical modifications performed to improve mRNA stability and biocompatibility with cells. Details on the IVT procedure, purification and quality control, nucleotide composition analysis, concentration determination, and purity assessment have been described [[16], [17], [18]]. Noteworthy, chemical modifications consisted of replacing 25 % of uridine residues with 2-thio-uridine, and 25 % of cytidine residues with 5-methyl-cytidine. In addition, a non-coding (NC) cmRNA was generated using a scrambled template of the Kozak sequence that was used as a control cmRNA. Sequences of cmRNAs used in this study can be found in the Dataverse repository (https://doi.org/10.34894/LAJVWD).

cmRNAs were formulated into lipid nanoparticles by using RmesFect™, 3D-FectIN™, and 3D-Fect™ (OZ Biosciences SAS, Marseille, France) as well as Lipofectamine™ MessengerMAX™ (hereafter termed as LipoMM, Invitrogen, Waltham, MA, USA). In addition to these well-established lipid vectors, the experimental lipids NL51 and NL37 (OZ Biosciences SAS) were investigated. A detailed description of the composition of the lipids is provided in the supplementary information file accompanying this article, with Table S1 summarizing the key differences between them. Formulated cmRNA lipoplexes were characterized for their morphology, hydrodynamic diameter, and zeta potential following the methodology described in supplementary information.

### 2.2 Transfection screening in hMSCs

Prior to experiments with hMSCs, studied lipids were titrated for protein expression and cytotoxicity using the immortalized human embryonic kidney 293 cell line (HEK293; DSMZ, Leibniz, Germany). Details can be found in supplementary information and in Fig. S1 and Fig. S2. The obtained results allowed a preliminary selection of mRNA concentrations and mRNA:lipid ratios to investigate further using hMSCs.

hMSCs were seeded in 96-well plates at a density of 15,000 cells/cm^2^. After 24 h, BMP-7 cmRNA complexes were prepared using pre-selected lipid vectors: RmesFect™ (1:4 ratio), NL51 (1:4 ratio), NL37 (1:4 and 1:5 ratios), 3D-FectIN™ (1:2 ratio), 3D-Fect™ (1:2, 1:4, and 1:5 ratios), and LipoMM (1:1 ratio). For all conditions, 0.125 μg of BMP-7 cmRNA was used per well, corresponding to 25 pg per seeded cell.

Complexes were freshly prepared in non-supplemented Opti-MEM and incubated for 20 min at room temperature to allow assembly and complex formation. The culture medium was removed from the hMSC-seeded plates, and 100 μL of the lipoplex solution was added to each well. After 6 h of incubation, the medium was replaced with fresh Opti-MEM (Life Technologies, Carlsbad, CA, USA) supplemented with 10% heat-inactivated Fetal Bovine Serum (FBS; Sigma–Aldrich, St. Louis, MO, USA) and 1% penicillin/streptomycin (P/S; 100 U/ml; Thermo Fisher Scientific, Waltham, MA, USA).

### 2.3 BMP-7 protein production

Supernatants were collected after days 1, 2, 3, 5, and 7 in new 96-well plates and stored at −80 °C. BMP-7 protein quantification was performed using the collected supernatants and the DuoSet ELISA kit (R&D Systems, Minneapolis, MN, USA) according to the manufacturer's instructions. Absorbance was measured at 450 nm and 540 nm using a CLARIOSTAR plate reader (BMG Labtech, Ortenberg, Germany). BMP-7 protein concentrations were calculated based on a standard curve and reported as pg protein/mL. Samples were measured in triplicate (n = 3).

### 2.4 Metabolic activity

The metabolic activity of hMSCs was assessed on days 1, 3, and 7 post-transfection using the commercial MTS assay (Abcam, Cambridge, UK). Briefly, 20 μL of MTS reagent was added to 80 μL of non-supplemented Opti-MEM per well, and the plates were incubated for 3 h under standard culture conditions. After incubation, the plates were briefly shaken to ensure homogeneity, and absorbance was measured at 490 nm using a CLARIOSTAR plate reader (BMG Labtech). Samples were measured in triplicate (n = 3).

### 2.5 Osteogenic gene expression in hMSCs

Transfection of hMSCs with BMP-7 cmRNA was performed as previously described to evaluate the expression of osteogenic-related genes using vectors NL37 (1:4 ratio) or LipoMM (1:1, ratio). These transfection conditions were selected based on the cmRNA and lipid titration performed in reporter HEK293 cells (supplementary information) and on the screening performed before this experiment using hMSCs.

To ensure sufficient RNA collection, cells were seeded in 12-well plates at a density of 25,000 cells/cm^2^. Five distinct donors of hMSCs were transfected independently. In addition, for each single cell donor, transfections were performed in triplicates. At days 3, 7, 14, 21, 28, and 35 post-transfection, cells were washed twice with Dulbecco's Phosphate-Buffered Saline (DPBS) and lysed using TRIzol reagent (Life Technologies). Total RNA was extracted using the phenol-chloroform method, with GlycoBlue (Invitrogen) used as a co-precipitant to enhance RNA recovery. RNA concentration and purity were assessed spectrophotometrically with the BioDrop μLITE (Biochrome, Cambridge, MA, USA).

First-strand cDNA synthesis was performed using 1 μg of total RNA and the iScript cDNA synthesis kit (Bio-Rad Laboratories Inc., Hercules, CA, USA), following the manufacturer's protocol. The expression levels of osteogenic genes (*RUNX2, ALPL, COL1A1, SPARC, SPP1*, and *BGLAP*) were quantified using real-time quantitative reverse transcription polymerase chain reaction (RT-qPCR). Details of the used primers can be found in the Dataverse repository ( https://doi.org/10.34894/LAJVWD).

RT-qPCR reactions were prepared using SYBR Green Master Mix (Bio-Rad Laboratories Inc.) and run on a Bio-Rad CFX96 real-time thermal cycler. Human beta-tubulin was used as the housekeeping gene for normalization. Relative gene expression levels were calculated using the 2^−ΔΔCt^ method, with untransfected cells serving as the control group.

### 2.6 Alkaline phosphatase activity

Alkaline phosphatase (ALP) activity in hMSCs was assessed using a colorimetric assay. Cells were seeded in 48-well plates at a density of 25,000 cells/cm^2^. After 24 h, they were transfected with BMP-7 cmRNA complexes prepared with NL37 (1:4 ratio) or LipoMM (1:1 ratio), as previously described. Independent transfections were performed using hMSCs from five distinct donors, with each donor condition tested in triplicate. At days 7, 14, 21, 28, and 35 post-transfection, cells were washed twice with DPBS and incubated with freshly prepared ALP substrate solution for 30 min at 37 °C. The substrate solution consisted of 3.5 mM 4-nitrophenyl phosphate disodium salt hexahydrate dissolved in 0.1 M ALP buffer (pH 10.5), which contained 50 mM glycine, 100 mM Tris-Base, and 2 mM MgCl_2_. A standard curve of 4-nitrophenol was prepared for quantification. After incubation, samples and standards were transferred to a new plate, and absorbance was measured at 405 nm using a CLARIOSTAR plate reader (BMG Labtech). A total of 15 samples per group (3 replicates × 5 donors) were analyzed for each time point.

To visualize specific ALP-positive areas, cells were washed with DPBS and fixed with 3.7% paraformaldehyde (PFA) for 10 min at room temperature. After removing the PFA, cells were incubated with an ALP staining solution containing 0.06% (w/v) Fast Blue B salt and 0.01% (w/v) Naphthol AS-MX phosphate for 30 min at 37 °C. The staining solution was subsequently removed, and cells were washed with DPBS. Stained cells were imaged using an inverted Nikon Ti-S/L100 microscope equipped with a Nikon DS-Ri2 camera (Nikon Europe, Amsterdam, the Netherlands) and a CoolLED pE100 system (CoolLED Limited, Andover, UK) for diascopic white light. A CFI Plan Apo LBDA 10X/0.45 objective (Nikon Europe) was used for imaging. Purple-stained areas were identified as ALP-positive regions.

### 2.7 Alizarin red staining

Calcium deposition in transfected hMSCs was assessed using Alizarin Red staining. Transfections were conducted as described before. At days 21, 28, 35, 42, and 49 post-transfection, cells were fixed with ice-cold 96% ethanol for 30 min. Following fixation, cells were washed twice with deionized water (dH_2_O) and stained with 0.5% Alizarin Red solution for 10 min at room temperature. Next, cells were thoroughly washed with dH_2_O to remove excess dye and imaged using the inverted Nikon Ti-S/L100 microscope (Nikon Europe). To quantify calcium deposits, dye from the stained regions was extracted with 10% hexadecylpyridinium chloride. Samples were incubated at room temperature for 15 min, after which the solubilized dye was transferred to a 96-well plate. Absorbance was measured at 562 nm using a CLARIOSTAR plate reader (BMG Labtech). Calcium concentration was calculated using an Alizarin Red dye standard curve and reported as mg/mL.

### 2.8 Immunostaining for osteopontin

Transfections were performed as previously detailed. At days 21, 28, 35, 42, and 49 post-transfection, cells were washed twice with DPBS and fixed with 3.7% PFA for 10 min at room temperature. Fixed cells were then incubated for 1 h at room temperature in a blocking solution containing 1% bovine serum albumin (BSA; Thermo Fisher Scientific), 10% normal goat serum (R&D Systems), 0.3 M glycine, and 0.1% Tween-20 in DPBS to minimize nonspecific binding. Following blocking, cells were incubated overnight at 4 °C with a primary rabbit anti-human osteopontin antibody (1:200; Abcam). After primary antibody incubation, cells were washed three times with DPBS and then incubated with an AlexaFluor568-conjugated secondary antibody (1:500; Thermo Fisher Scientific) for 1 h at room temperature in the dark. Cell nuclei were counterstained with 4′,6-diamidino-2-phenylindole (DAPI; Thermo Fisher Scientific). Samples were then imaged to assess osteopontin expression.

### 2.9 Proteomic analysis of hMSCs transfection

To investigate early protein abundance changes following BMP-7 cmRNA transfections, bottom-up label-free proteomics was employed. hMSCs were transfected as previously described using NL37 (1:4 ratio) or LipoMM (1:1 ratio). As control groups, cells unstransfected or transfected with NC cmRNA were included. In the case of the transfected control group, identical transfection conditions and lipids were used. For sufficient protein yield, hMSCs were seeded in 6-well plates at a density of 25,000 cells/cm^2^. Transfections were performed using cells from five distinct hMSC donors on independent experiments. Following transfection, the medium was replaced with serum-free Opti-MEM.

At 48 h post-transfection, cells were harvested using ammonium acetate precipitation with a 150 mM solution. The cell pellets were resuspended in a 5 M urea buffer, prepared by diluting 5 M urea (GE Healthcare, Chicago, IL, USA) in 50 mM ammonium bicarbonate buffer. To ensure effective protein extraction, three freeze-thaw cycles were performed on the harvested cells. The protein extracts from all five donors were pooled for each condition.

#### 2.9.1 In-solution digestion

Protein concentrations were determined using a Bradford-based protein assay. Briefly, 200 μL of Quick Start™ Bradford 1X Dye Reagent was added to each well of a 96-well plate containing either the standard solution (1.35 mg/mL BSA) or the sample. The plate was incubated at room temperature for 5 min, followed by 15 s of shaking. Absorbance was measured at 595 nm using a CLARIOSTAR plate reader (BMG Labtech). All standards and samples were analyzed in duplicate. A total of 30 μg of protein was dissolved in 30 μL of 50 mM ammonium bicarbonate (ABC) containing 5 M urea. To reduce the proteins, 3 μL of dithiothreitol (DTT, 20 mM final concentration) was added, and the mixture was incubated at room temperature for 45 min.

Following reduction, the proteins were alkylated by adding 4 μL of iodoacetamide (IAA, 40 mM final concentration). The reaction was performed in the dark at room temperature for 45 min. Alkylation was quenched by adding 6 μL of DTT (to consume unreacted IAA), followed by an additional incubation at room temperature for 45 min.

For protein digestion, 2 μg of trypsin/lysC was added to the sample and incubated at 37 °C for 2 h. To dilute the urea concentration, 120 μL of 50 mM ABC was added, and the digestion was continued at 37 °C for 18 h. After digestion, the mixture was centrifuged at 2500 × g for 5 min, and the supernatant was collected for further analysis.

#### 2.9.2 Protein identification by liquid chromatography–mass spectrometry (LC-MS)

A nanoflow high-performance liquid chromatography (HPLC) system (Dionex Ultimate 3000, Thermo Fisher Scientific) was coupled on-line to a Q Exactive mass spectrometer (Thermo Fisher Scientific) equipped with a nano-electrospray Flex ion source (Proxeon, Thermo Fisher Scientific). A 5 μL aliquot of the peptide digest mixture was loaded onto a reversed-phase C18 column (Acclaim PepMap C18, 75 μm inner diameter × 50 cm length, 2 μm particle size, Thermo Fisher Scientific). Peptide separation was achieved using a 240-min linear gradient of 4–45% buffer B (80% acetonitrile, 0.08% formic acid) at a flow rate of 300 nL/min.

Mass spectrometry data were acquired using a data-dependent Top 10 method in positive ion mode. Survey scans were performed over an *m*/*z* range of 250–1250 at a resolution of 70,000 with a maximum injection time of 100 ms. The dynamic exclusion duration was set to 30 s to minimize repeated measurements of the same precursor ions.

Precursor ions were isolated using a 2.0 *m*/*z* window, and the most abundant ions were selected for fragmentation via high-energy collision dissociation. Fragment spectra were acquired at a resolution of 17,500 with a normalized collision energy of 30 eV and a maximum injection time of 200 ms. The underfill ratio was set to 1.0%.

Peptide recognition mode was enabled, excluding singly charged ions and ions with charge states greater than five.

#### 2.9.3 Database search and quantification

The mass spectrometry data were searched using Proteome Discoverer 2.2 Sequest HT search engine (Thermo Fisher Scientific), against the UniProt human database. The false discovery rate (FDR) was set to 0.01 for proteins and peptides, which had to have a minimum length of 6 amino acids. The precursor mass tolerance was set at 10 ppm and the fragment tolerance at 0.02 Da. One miss-cleavage was tolerated; oxidation of methionine was set as a dynamic modification. Carbamidomethylation of cysteines was set as fixed modification. Label free quantitation was conducted using the Minora Feature Detector node in the processing step and the Feature Mapper node combined with the Precursor Ions Quantifier node in the consensus step with default settings within Proteome Discoverer 2.2. Normalization was done against the total peptide amount per sample.

Quantified proteins were analyzed based on their abundances, abundance ratios relative to the control groups (i.e., untransfected and NC cmRNA transfected hMSCs), and p-values, with a significance threshold set at *p* ≤ 0.05. Proteins were classified as up- or down-regulated based on their fold change values. Proteins in volcano plots are displayed by statistical significance (y-axis, -log10 *p-value*) and relative abundance (x-axis, log2 fold change). Orange dots represent significantly downregulated proteins, while blue dots indicate significantly upregulated proteins. The heatmap shows abundance values, expressed as fold changes normalized to the control groups.

Functional protein association networks, along with enrichment analyses for molecular functions, cellular components, and Reactome pathways, were generated using the STRING database, string-db.org [39]. The analysis was conducted separately for up- and down-regulated protein networks for hMSCs transfected with each lipid vector investigated. In addition, to complement the data, the online tool Metascape was also used to obtain enriched terms and pathways http://metascape.org [40].

### 2.10 Subcutaneous implantation

In our study, 88, eight-week-old female nude mice (BALB/cOlaHsd-Foxn1nu; Envigo RMS B.V., Melderslo, the Netherlands) were used. Animals were assigned to five experimental groups, comprising both control and treatment conditions. To assess potential dose-dependent effects, three concentrations of BMP-7 cmRNA were evaluated. The groups included: (i) scaffolds seeded with hMSCs alone (UNS; control), (ii) scaffolds seeded with hMSCs and NC cmRNA lipoplexes (NC; control), and (iii) scaffolds seeded with hMSCs and low (0.036 μg), medium (0.36 μg), or high (3.6 μg) doses of BMP-7 cmRNA. Detailed information on the animals and ethical approval, as well as the protocol used for the preparation of scaffolds and transfection complexes for *in vivo* implantation, can be found in the supplementary information.

Approximately 1 h prior to surgery, animals received subcutaneous injections of buprenorphine (0.1 mg/kg) for preoperative analgesia. Anesthesia was induced with 5% isoflurane and maintained at 1.5% via inhalation. During the procedure, animals were positioned prone on a heated platform covered with absorbent material to maintain body temperature. Isoflurane was continuously administered via a nose cone to ensure the animals remained anesthetized throughout the procedure. The dorsal skin was disinfected with 70% chlorhexidine and 70% ethanol. For each implantation site, a 4 mm incision was made along the back, parallel to the spine, using surgical scissors. Blunt dissection was then performed to separate the skin from the underlying subcutaneous connective tissue, creating pockets for implantation. Up to four scaffolds were randomly implanted per animal, with two pockets on each side of the spine (one cranial and one caudal). The incisions were closed with monofilament sutures. Following the procedure, a subcutaneous injection of carprofen (10 mg/kg) was administered for postoperative pain management. Mice were monitored closely until they fully recovered mobility and awareness. To ensure continued analgesia, carprofen was also provided in the drinking water for three days post-surgery.

To assess early outcomes, we evaluated human BMP-7 production via ELISA and examined the biodistribution of implanted cmRNA using RT-qPCR at days 1 and 3 post-implantation (timeline schematic in Fig. S3a). To monitor bone regeneration over time, *in vivo* μCT imaging was conducted at weeks 3, 5, and 7. In parallel, we performed gene expression analysis, histological staining, and immunohistochemistry at weeks 1, 2, 4, and 8 to investigate osteogenesis, vascularization, and innervation (Fig. S3b).

### 2.11 Human BMP-7 expression

On postoperative days 1 and 3, mice were euthanized, and the implanted scaffolds were surgically retrieved. The extracted scaffolds were immediately placed in 2 ml Precellys tubes (Bertin Instruments, Ile-de-France, France), snap-frozen in liquid nitrogen, and stored at −80 °C for further analysis. To assist with tissue homogenization, three 2.38 mm metallic beads (Qiagen, Hilden, Germany) were added to each tube. The tubes were then processed in the Precellys® 24 Touch homogenizer (Bertin Instruments) for an initial homogenization cycle of 60 s at 6000 rpm. Following this, 500 μl of T-PER tissue protein extraction solution (Thermo Fisher Scientific), supplemented with a protease inhibitor cocktail, was added to each tube, and a second homogenization cycle was performed under the same conditions. Samples were then centrifuged at 10,000 x g for 5 min at 4 °C. The supernatant, containing the extracted proteins, was carefully collected and transferred to fresh tubes. Total protein concentration was measured using a BCA assay kit, following the manufacturer's instructions (Thermo Fisher Scientific). Human BMP-7 levels were quantified using DuoSet ELISA kits in accordance with the manufacturer's instructions. Absorbance was recorded at 450 nm and 540 nm with a CLARIOSTAR plate reader (BMG Labtech). Readings at 540 nm were subtracted from readings at 450 nm to correct for optical imperfections in the plate. BMP-7 protein concentrations were calculated from a standard curve, and results were reported in pg of BMP-7/mg of total protein, with n = 9 biological replicates.

### 2.12 cmRNA localization in different organs

On days 1 and 3 post-surgery, mice were euthanized, and the lungs, spleen, liver, and kidneys were surgically extracted. These organs were immediately placed into 2 ml Precellys tubes, snap-frozen in liquid nitrogen, and stored at −80 °C for subsequent analysis. Tissue homogenization was carried out using the Precellys® 24 Touch homogenizer, following a similar procedure as before. However, in this case, 500 μl of TRIzol reagent was added to each tube instead of the T-PER buffer with protease inhibitors.

Total RNA was extracted using the phenol/chloroform method, with GlycoBlue (Invitrogen) added as an RNA co-precipitant. RNA concentration and purity were assessed spectrophotometrically using the BioDrop μLITE (Biochrome). First-strand cDNA was synthesized from total RNA using the iScript cDNA Synthesis Kit (Bio-Rad Laboratories Inc.) following the manufacturer's instructions. Expression levels specific for the BMP-7 cmRNA were quantified by RT-qPCR. Primer sequences used for amplification are listed in the Dataverse repository ( https://doi.org/10.34894/LAJVWD). Absolute gene expression levels in RNA samples collected from various organs were quantified by interpolating Ct values against a standard curve generated from serial dilutions of BMP-7 cmRNA (starting from 1 ng/μL in 10 μL total volume). The dilution series included 1:10, 1:100, 1:500, 1:1000, 1:2000, and 1:5000, with a constant reaction volume maintained across samples. The standard curve was based on 12 measurements (6 biological replicates × 2 technical replicates).

### 2.13 In vivo μCT analysis

At weeks 3, 5, and 7 post-implantation, bony tissue formation within the implants was evaluated through *in vivo* micro-computed tomography (μCT) imaging. Mice were anesthetized with 4% isoflurane, which was maintained at 2% throughout the scans using a nose cone within the μCT (Fig. S4). Scans were performed using a small animal μCT (X-RAD SsmART 225Cx, Precision X-ray Inc., North Branford, CT, USA), operating at 80 kVp and 2.5 mA with a 2 mm aluminum filter. Imaging was conducted during a 360° rotational scan with an acquisition rate of 5 frames per second and a gantry rotation speed of 1 revolution per minute. Bone volume quantification was normalized to the volume of each scaffold (BV/TV). Data were processed using threshold-based segmentation and 3D analysis. Renderings of the structures were generated using 3D Slicer software (version 5.6.1, http://www.slicer.org) [41]. A total of 6 samples per group were scanned for analysis.

### 2.14 Osteogenic PCR array

Expression of osteogenesis-related genes was evaluated at weeks 1, 2, 4 and 8 post-implantations. Explants were collected in 2 ml Precellys tubes, rapidly frozen in liquid nitrogen, and stored at −80 °C. Tissue homogenization was performed using the Precellys® 24 Touch homogenizer with 500 μl of TRIzol, following the previously outlined protocol. Total RNA was extracted using the phenol/chloroform method, as described earlier. RNA concentration and purity were measured spectrophotometrically with the BioDrop μLITE (Biochrome). First-strand cDNA synthesis was carried out using the RT^2^ First Strand Kit (Qiagen) according to the manufacturer's instructions. The RT^2^ Profiler™ PCR Array for Mouse Osteogenesis (GeneGlobe Id: PAMM-026ZD, Qiagen) was performed using RT^2^ SYBR Green Master Mix on a Bio-Rad CFX96 thermal cycler (Bio-Rad Laboratories Inc.), following the recommended protocol. A list of the 84 measured genes is provided in Table S2 (supplemental Excel file). This array evaluated the expression of growth factors, transcription factors, and extracellular matrix components associated with skeletal development and bone mineral metabolism. The Ct values were uploaded to the GeneGlobe data analysis web portal (www.qiagen.com/geneglobe) for processing. Heat maps indicated the magnitude of gene expression, calculated by 2^–ΔCT^ for each gene and normalized to the average 2^–ΔCT^ across all samples. Bar graphs displayed the fold changes in up- and downregulated genes using the 2^−ΔΔCT^ formula. Scatter plots compared normalized gene expression between treatment groups and the NC control group to quickly visualize large gene expression changes.

### 2.15 Histology

At weeks 1, 2, 4, or 8 post-implantations, a separate set of scaffolds were explanted and placed in 15 ml Falcon tubes containing 1% paraformaldehyde (PFA) in PBS. To ensure optimal fixation, the PFA solution was freshly prepared daily from powdered PFA. Samples were fixed overnight at 4 °C, followed by a transfer to fresh PBS to remove any remaining fixative. For decalcification, each sample was immersed in 10 ml of a solution consisting of 7% EDTA (w/v) and 10% sucrose (w/v) in PBS, which was replaced daily over the course of a month to maintain effectiveness. Samples were incubated at 37 °C throughout the decalcification process. Once decalcified, the samples were embedded in molds with optimal cutting temperature compound (O.C.T. Tissue-Tek®, Sakura, Torrance, CA, USA) and slowly frozen by immersing the molds in isopentane placed in a vessel with liquid nitrogen. The frozen samples were stored at −80 °C. Cryosections were prepared by slicing the samples into 10 μm sections using a Leica CM3050 cryostat (Leica Biosystems, Nussloch, Germany), with the chamber temperature set at −20 °C.

Histological analysis was carried out using the Movat Pentachrome Stain Kit (Modified Russell-Movat, Abcam) as per the manufacturer's instructions. This staining method colors elastic fibers and nuclei black/blue, collagen yellow, mucin bright blue, fibrin bright red, and muscle red. Brightfield imaging of the entire scaffold and selected magnified areas was performed using a Nikon Ti-E automated inverted microscope (Nikon Europe) equipped with a Spectra light source (Lumencor, Beaverton, OR, USA) and an Andor Zyla 5.5 sCMOS camera (Oxford Instruments, Abingdon, UK). A CFI Plan Apochromat K 4X NA 0.2 WD 20 objective (Nikon Europe) was used for imaging. The yellow-stained collagen areas were quantified using Fiji software (https://fiji.sc/).

### 2.16 Immunostainings

Sectioned scaffolds were incubated with 1% BSA, 10% normal goat serum, 0.3 M glycine, in 0.1% DPBS-Tween for 1 h to permeabilize cells and block non-specific protein-protein interactions. Separate samples were incubated with either anti-collagen type 1 (1:2000, Abcam), anti-osteopontin (1:500, Invitrogen), or anti-ostecalcin (1:300, Abcam) primary antibodies for 24 h at 4 °C. Sections were washed 3 times with DPBS and incubated with species-specific secondary antibodies conjugated to Alexa Fluor 647 (1:2000). Cell nuclei were stained with DAPI (1:1000). Osteocalcin stained samples were additionally stained with Phalloidin 488 (1:300) to visualize actin filaments. Secondary antibodies, DAPI, and phalloidin were all obtained from Thermo Fisher Scientific. Fluorescent imaging of the complete scaffold and magnified areas was performed using the same inverted Nikon Ti-E microscope. The objective CFI Plan Fluor 40x Oil A NA 1.3 WD 0.2 mm was used for the magnified areas (Nikon Europe). Quantification of the area positive to OPN and COL1A1 on the acquired images took place using the Fiji software (https://fiji.sc/, [42]).

Immunofluorescence staining for angiogenesis analysis was performed using a rat anti-mouse CD31 antibody (clone MEC 13.3, BD Bioscience, San Jose, CA, USA) at 1:100 dilution, followed by fluorescently labeled secondary antibodies (1:200, Thermo Fisher Scientific). Images were acquired using a Nikon Ti2 Eclipse microscope (Nikon Europe). Vascular density was analyzed at weeks 1, 2, 4, and 8 post-implantation by quantifying CD31 signal using QuPath v0.4.4 https://qupath.github.io/, [43]. Whole-section reconstructions were generated, and CD31-positive pixels were quantified to assess vascularization.

For assessment of innervation, antigen retrieval was performed by incubating sections in citrate buffer (Target Retrieval Solution, Low pH - Dako Omnis®, Agilent Technologies, Santa Clara, CA, USA) at 95 °C for 20 min for, followed by permeabilization with 0.5% Triton X-100 in PBS (pH 7.4) for 30 min at room temperature. Blocking was performed using 0.1% Triton X-100 and 10% sheep serum in PBS for 1.5 h at room temperature. Sections were then incubated overnight at 4 °C with anti β3-tubulin (1:1000, Abcam) in blocking buffer. The next day, sections were incubated for 1.5 h at room temperature with an Alexa Fluor 568-conjugated anti-rabbit IgG secondary antibody (1:500, Invitrogen) in PBS. Cell nuclei were counterstained with DAPI (1:1000, Thermo Fisher Scientific) for 20 min at room temperature. Samples were then mounted using Aqua-Polymount (Polysciences®, VWR, West Chester, PA, USA). Sections were imaged using a confocal laser scanning microscope (SP8, Leica Microsystems, Wetzlar, Germany) with a 20X objective. All areas were determined based on tissue autofluorescence and created manually with the Imaris® “Surface” tool.

### 2.17 Statistical analysis

Statistical analyses were performed using GraphPad Prism (version 10.2.3; GraphPad Software, San Diego, CA, USA). The normality of data distribution was evaluated using the Shapiro-Wilk test and the D'Agostino-Pearson test. For comparisons across multiple groups, a two-way analysis of variance (ANOVA) followed by appropriate post-hoc tests was applied. Dunnett's multiple comparison test was used for BMP-7 protein quantification, ALP and Alizarin Red quantifications, MTS assays, and quantification of CD31 density and vascular invasion. Šídák's multiple comparison test was employed for analyzing gene expression, size, and zeta potential measurements, and μCT measurements of BV/TV ratios. Tukey's multiple comparison test was applied to osteopontin quantification, BMP-7 protein levels *in vivo*, and quantification of stained areas of explants. A *p*-value of less than 0.05 was considered statistically significant, with p-values reported as ∗*p* ≤ 0.05, ∗∗*p* ≤ 0.01, ∗∗∗*p* ≤ 0.001, and ∗∗∗∗*p* ≤ 0.0001.

## 3 Results

### 3.1 Optimal lipid vector and transfection conditions drive robust BMP-7 production *in vitro*

Lipid materials have been commonly used to internalize mRNA inside cells. It is, however, well acknowledged that their efficiency and biocompatibility highly depend on the lipid's own structure, and often do not go hand in hand. In our study, we screened 6 lipids by using HEK293 cells. Protein production and cytotoxicity in the reporter cell line indicated 25 pg cmRNA/cell on a 1:1 – 1:5 mRNA:Lipid ratio range as adequate. Fig. S5 illustrates the protein production results, while Fig. S6 shows cytotoxicity after transfection. Data are further explained in the results section of the supplementary information material. Based on the screening results, the best-performing transfection conditions were selected for a more directed investigation in hMSCs using BMP-7 cmRNA.

**Fig. 1:**
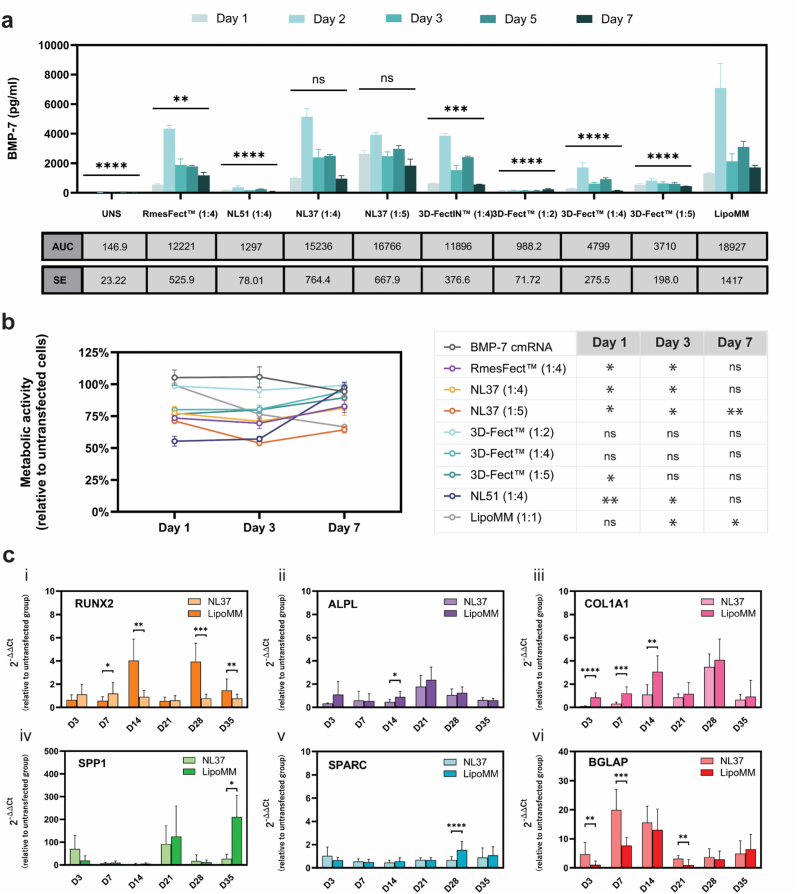
| Selection of optimized lipid vectors for BMP-7 cmRNA delivery. (**a**) BMP-7 protein production in transfected hMSCs, highlighting superior performance of NL37 and LipoMM vectors. Statistical analysis was performed to compare conditions against LipoMM. (**b**) Metabolic activity of transfected hMSCs assessed via MTS assay, showing cellular viability across conditions relative to the control group (BMP-7 cmRNA only, no lipid). **(c)** Osteogenic gene expression post-transfection**.** (**i**) RUNX2: Modest upregulation at day 7 with NL37 and strong expression at days 14, 28, and 35 with LipoMM. (**ii**) ALPL: Significantly higher levels at day 14 post-transfection with LipoMM. (**iii**) COL1A1: Gradual increase over time in both groups, with significantly higher levels at days 3, 7, and 14 in the LipoMM group compared to NL37. (iv) SPP1: Strong upregulation at days 3, 21, and 35. (v) SPARC: Modest upregulation at day 28 in the LipoMM group. (**vi**) BGLAP: Significantly higher expression at days 3, 7, and 21 in the NL37 group compared to LipoMM. Data are presented as mean ± SD (in a and b, n = 3; in c, n = 15 (5 hMSC donors × 3 replicates)). Multiple comparisons were analyzed using two-way ANOVA with Dunnett's correction in (A) and (B) and Šídák's correction in (C) and (D) ∗p < 0.05, ∗∗p < 0.01, ∗∗∗p < 0.001, ∗∗∗∗p < 0.0001. Abbreviations: AUC, area under the curve; SE, standard error.

BMP-7 production by transfected hMSCs was observed across all transfection conditions over a 7-day period, with peak levels occurring on day 2 post-transfection (Fig. 1a). Transfections with LipoMM complexes yielded the highest BMP-7 levels over time, with an area under the curve (AUC) of 18,927 ± 1417 and a protein production peak of 7095.9 ± 1678 pg/ml at day 2. Similar results were obtained for experimental lipid NL37. This lipid vector resulted in a BMP-7 expression not significantly different from LipoMM (NL37 [1:4 mRNA:Lipid]: AUC 15,236 ± 764.4, *p = *0.132; NL37 [1:5 mRNA:Lipid]: AUC 16,766 ± 667.9, *p = *0.8775). NL37 protein production peak was 5150 ± 561 pg/ml (1:4 mRNA:Lipid) and 3926 ± 162 pg/ml (1:5 mRNA:Lipid) at day 2.

Vectors RmesFect™, 3D-FectIN™, and 3D-Fect™, along with the newly designed NL51 showed significantly lower protein expression, up to 15-fold reduction, when compared to LipoMM and NL37. AUC values were 12,221 ± 525.9 for RmesFect™, 11,896 ± 376.6 for 3D-FectIN™, and 1297 ± 78.01 for NL51. The three different mRNA:Lipid ratio tested for 3D-Fect™ resulted in AUC range from a maximum of 988.2 ± 71.72 to a minimum of 4799 ± 275.5.

### 3.2 High cmRNA-to-lipid ratios and cmRNA concentrations reduce metabolic activity

Metabolic activity, normalized to untransfected cells, revealed varying cytotoxic profiles among the tested vectors (Fig. 1b; Naked BMP-7 cmRNA used as a control). Although LipoMM showed initial good, ∼100%, cell viability (day 1; Fig. 1b), the toxicity of this lipid observed in HEK293 (Fig. S6) was confirmed in hMSCs. A constant decline in cell viability was observed post-transfection, which reached 67% by day 7 (*p < *0.05 vs. control; Fig. 1b). Given its high protein expression efficiency, the next interesting lipid was NL37. NL37 (1:4 ratio) resulted in ∼75% metabolic activity at day 1, with a modest 5% reduction by day 3 (p < 0.05 vs. control). Interestingly, cells recover, and by day 7, an approximate 80% cell viability was obtained. NL37's compatibility depended on the mRNA:Lipid ratio used, with a decrease in cell viability accompanying the increased ratio. The rest of the lipids evaluated showed adequate cell viability (>80%), which, similarly to NL37, depended on the ratio used. Higher ratios reduced metabolic activity by ∼25% (e.g., 1:4, 1:5 for 3D-Fect™; Fig. 1b). Worth noticing is the initial toxicity that complemented the use of NL51 as a lipid vector. A ∼55% metabolic activity was obtained for the first 48 h after transfection with this lipid (*p = *0.0026).

Based on BMP-7 protein expression and cell viability, NL37 (1:4 ratio) and LipoMM were selected for further studies. Although NL37 at a 1:5 ratio showed slightly higher protein expression (AUC 11,947 vs. 11,766), the 1:4 ratio was favored due to lower cytotoxicity. Despite its toxicity, LipoMM was included as a widely used reference with established efficacy in the literature. The physicochemical properties of BMP-7 cmRNA/NL37 and BMP-7 cmRNA/LipoMM lipoplexes, including size, electrokinetic potential, and morphology, are depicted in Fig. S7. Notably, NL37 lipoplexes displayed a positive electrokinetic potential, in contrast to LipoMM, which formed negatively charged complexes. Both systems yielded particles of roughly 200 nm; however, incubation in cell culture medium caused a substantial size increase in NL37 complexes to approximately 800 nm.

### 3.3 BMP-7 cmRNA induces osteogenic gene expression

Transfection with BMP-7 cmRNA lipoplexes induced distinct osteogenic gene expression profiles (Fig. 1c). Runt-related transcription factor 2 (RUNX2) showed an early, moderate increase with NL37 complexes (days 3, 7), significantly higher than LipoMM at day 7 (*p = *0.0423). Conversely, LipoMM drove significantly higher RUNX2 expression at later time points (days 14, 28, 35; *p < *0.01). Alkaline phosphatase (ALPL) was moderately overexpressed, peaking at day 21 for both lipoplexes, but LipoMM showed significantly higher levels at day 14 (*p = *0.0205). Collagen type I alpha 1 chain (COL1A1) progressively increased, peaking at day 28. LipoMM consistently resulted in superior COL1A1 levels (*p < *0.0001, days 3, 7, 14). Secreted protein acidic and cysteine-rich (SPARC), also known as osteonectin, remained largely unchanged, except for a significant increase with LipoMM at day 28 (*p < *0.0001 vs. NL37).

Secreted phosphoprotein 1 (SPP1), also known as osteopontin, exhibited the most pronounced expression (up to 200-fold increase). NL37 elevated SPP1 at day 3, while LipoMM induced higher expression at days 21 and 35, with the most substantial difference at day 35 (*p < *0.0113). Bone gamma-carboxyglutamate protein (BGLAP), also known as osteocalcin, showed marked overexpression at early time points, with significantly higher levels from NL37 transfections compared to LipoMM on days 3, 7, and 21 (*p < *0.0021).

### 3.4 BMP-7 cmRNA stimulates ALP activity and mineralization

**Fig. 2:**
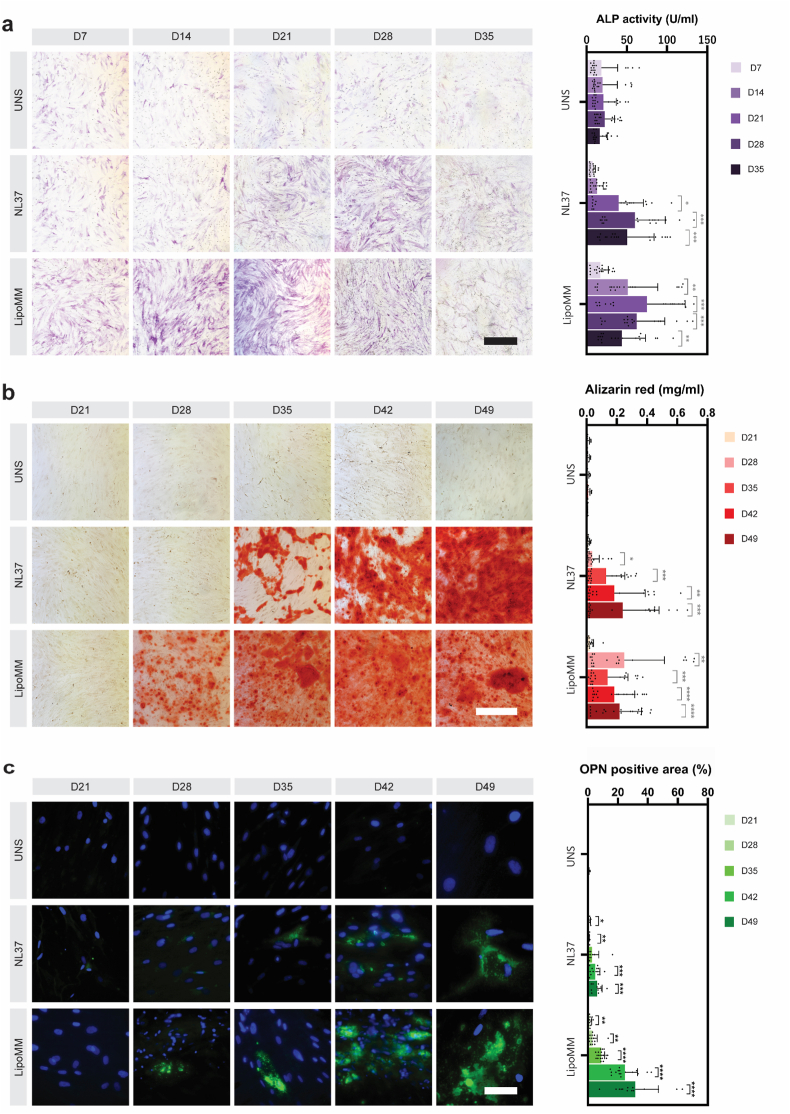
| Panels display osteogenesis and mineralization of hMSC transfected BMP-7 cmRNA. (**a**) Representative images of alkaline phosphatase (ALP) staining at days 7, 14, 21, 28, and 35 post-transfection, with corresponding colorimetric quantification. (**b**) Representative images of Alizarin Red S staining showing calcium deposition at days 21, 28, 35, and 42 post-transfection, alongside quantification of dissolved Alizarin Red S. (**c**) Fluorescence microscopy images of hMSCs at days 21, 28, 35, and 42 post-transfection, with nuclei stained by DAPI (blue) and osteopontin (OPN) staining (green). The graph quantifies the percentage of OPN-positive areas based on post-imaging analysis. Data are presented as mean ± SD (n = 15). Scale bars: 500 μm for (A) and (B), 50 μm for (C). Statistical analysis: two-way ANOVA with Dunnett's correction for (A) and (B), and Tukey's correction for (C). ∗p < 0.05, ∗∗p < 0.01, ∗∗∗∗p < 0.0001.

Transfection with either lipid vector carrying BMP-7 cmRNA resulted in a significant enhancement of alkaline phosphatase (ALP) activity in hMSCs (Fig. 2a, Fig. S8, Fig. S9). Both formulations induced early ALP activity, with increases becoming detectable from day 14 onward. NL37-mediated transfection generally exhibited a later peak (days 28–35), whereas earlier increases were observed in some LipoMM conditions (ALP activity quantification depicted in Fig. 2a). Donor-dependent variability was evident, as hMSC cultures displayed distinct basal ALP activities (Fig. S8, Fig. S9).

Alizarin Red staining and quantification confirmed calcium deposition in hMSCs upon transfection with BMP-7 cmRNA (Fig. 2b, Fig. S10, Fig. S11). Consistent with the ALP activity profiles, mineralization onset varied slightly between the two lipid formulations, with some cultures exhibiting detectable calcium deposition by day 28 (LipoMM) and others closer to day 35 (NL37). Despite these temporal differences, BMP-7 cmRNA/NL37-transfected cells frequently showed more pronounced calcium accumulation at later stages. Donor-dependent variability of calcium deposition upon transfection is shown in Fig. S10 and Fig. S11.

### 3.5 Osteopontin deposition upon BMP-7 cmRNA transfection correlates with mineralization

hMSCs transfected with BMP-7 cmRNA complexes revealed significantly increased osteopontin (OPN) expression in both NL37 and LipoMM groups, compared to the untransfected control group (green color; Fig. 2c). OPN deposition, as a result of BMP-7 cmRNA transfections, was detected in all used hMSCs donors (Fig. S12 and 13). OPN-positive areas were generally more abundant in the LipoMM transfection groups. This is particularly noticeable for days 35, 42, and 49 post-transfection.

### 3.6 Proteins relevant for intracellular vesicle transport, cytoskeletal changes, ECM, and calcium-regulation were upregulated after BMP-7 cmRNA transfer, mostly by NL37 delivery

**Fig. 3:**
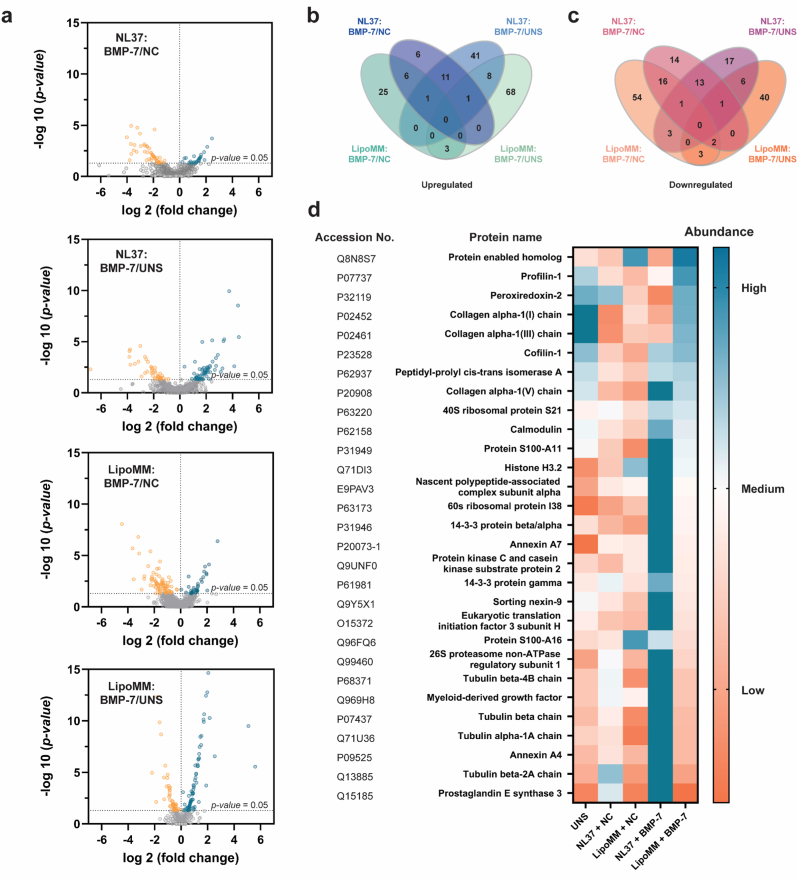
| Label-free quantitative proteomic analysis of early protein changes post-transfection. (**a**) Volcano plots illustrate significantly differentially expressed proteins identified through label-free quantitative proteomics in cell lysates collected 2 days post-transfection. Proteins are ranked according to their statistical p-value (y-axis) as -log 10 and their relative abundance ratio (log 2 fold change). Orange and blue dots depicts significantly downregulated and upregulated proteins, respectively. (**b**) and (**c**) show Venn diagrams depict the number of significantly expressed proteins unique to or shared among different experimental groups. (**d**) Heatmap visualizes major changes in protein abundance across groups: untransfected hMSCs, hMSCs transfected with non-coding cmRNA, or hMSCs transfected with BMP-7 cmRNA. Protein abundance is color-coded, with low, medium, and high values corresponding to levels of 20, 90, and 160, respectively. Transfections were performed using the lipid vectors NL37 or Lipofectamine™ MessengerMAX™.

Proteomic analysis identified 1287 proteins in hMSCs after BMP-7 cmRNA transfection, including 170 significantly upregulated and 168 significantly downregulated proteins. Volcano plots (Fig. 3a) illustrate the significantly differentially expressed proteins in cell lysates collected two days post-transfection with BMP-7 cmRNA delivered using NL37 or LipoMM vectors. Comparisons were made against NC cmRNA transfections and untransfected cells. Venn diagrams (Fig. 3b and c) show the overlap and unique protein expressions across experimental groups, while the heatmap (Fig. 3d) highlights the most significantly overexpressed proteins in BMP-7 cmRNA-transfected groups.

A differential protein regulation pattern was observed between the two lipid vectors used, with each inducing a distinct proteomic signature. Proteins upregulated by one formulation were generally low or minimally expressed in the other, and vice versa, highlighting non-overlapping mechanistic responses to NL37 and LipoMM (Fig. 3d). Upregulated proteins included those associated with extracellular matrix (ECM) production, such as collagen alpha-1 chains. Interestingly, BMP-7 cmRNA delivered via NL37 induced upregulation of collagen alpha-1(V), while delivery via LipoMM was associated with increased expression of type I and III collagen chains. Proteins crucial for cytoskeletal dynamics and organization, including cofilin-1 and profilin-1 were also upregulated; cofilin-1 was similarly influenced by both lipoplexes while profilin-1 was specifically upregulated by the BMP-7 cmRNA/LipoMM complexes. Tubulins (i.e., alpha-1A as well as beta-2A and -4B chains) were solely upregulated by BMP-7 cmRNA delivered via NL37. Also specific to NL37-mediated mRNA transfer, proteins associated with endocytosis and intracellular vesicle-mediated transport, such as protein kinase C and sorting nexin-9, were upregulated, alongside marked increases in calcium-binding regulators, including calmodulin-1, annexin A4, and protein S100-A11. Comprehensive details of statistically up- and downregulated proteins, including UniProt accession codes, abundance ratios (log2), and p-values, are presented in Table S3.

### 3.7 Differential signaling pathways underlie osteogenic outcomes of BMP-7 cmRNA

The differential protein regulation induced by the two lipoplexes was further reflected in their activated pathways, with protein interaction network analysis revealing distinct sets of significantly up- and downregulated pathways for both NL37 and LipoMM transfections (Fig. S14 and Fig. S15). Comparisons between BMP-7 cmRNA and NC cmRNA transfected cells identified functions and pathways affected uniquely by the produced BMP-7.

NL37-mediated cmRNA delivery activated cytoskeletal constituents, with enriched cellular components including fibrillar collagen trimers, microtubules, and extracellular exosomes (Fig. S14b, i-ii). Pathways related to GLUT4 translocation, AMPK activation, the HSP90 chaperone cycle, and tubulin folding were enriched (Fig. S14b, iii). Conversely, downregulated functions included TAP and peptide antigen binding, impacting components like phagocytic vesicles and the MHC class I complex (Fig. S14c). Downregulated pathways included endosomal processes and interferon signaling (Fig. S14d, i-iii).

cmRNA delivery performed with LipoMM upregulated PDGF binding, ECM structural support, and cell adhesion molecule binding (Fig. S15b and i). Enriched cellular components overlapped with NL37 (e.g., fibrillar collagen trimers, extracellular exosomes) but uniquely included focal adhesions and the cell leading edge (Fig. S15b, ii). Pathways involved syndecan interactions, collagen chain trimerization, ECM proteoglycans, and platelet degranulation (Fig. S15b, iii). Metascape analysis highlighted axon guidance, VEGFA/VEGFR2 signaling, and platelet activation (Fig. S16b). LipoMM downregulated TAP binding and, uniquely, pathways related to cornified envelope formation (Figs. S15c, d, i-iii). This divergence underscores the vector's influence on BMP-7-mediated cellular processes.

### 3.8 Dose-dependent ossification induced by BMP-7 TAMs in nude mice

**Fig. 4:**
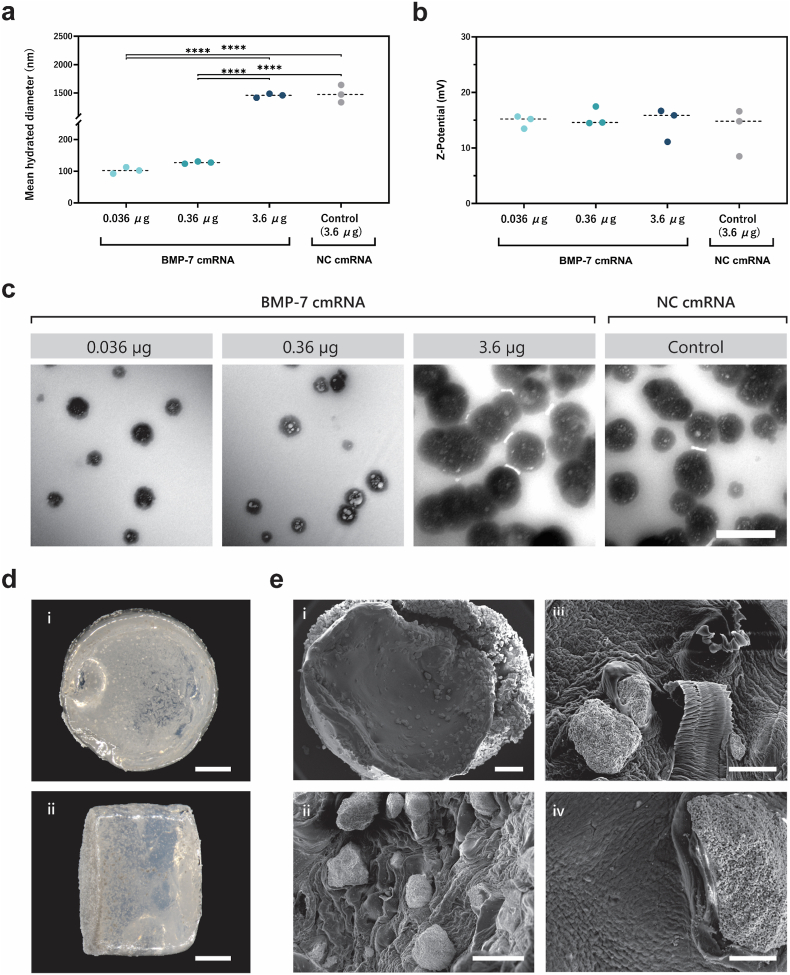
| Characterization of BMP-7 cmRNA complexes and fibrin-CaP scaffold. (**a**) Size displayed as mean hydrated diameter and (**b**) electrokinetic potential of BMP-7 cmRNA complexed with the lipid vector NL37. (**c**) TEM photomicrographs show morphological features of the lipid complexes. (**d**) Stereomicroscope images of top view (**i**) and side view (**ii**) of the fibrin-CaP scaffolds. (**e**) SEM images of the scaffold's overview (**i**) and magnified areas (**ii-iv**). Scale bar: (c) 500 nm, (d) 2 mm, (e) i, 500 μm; ii, 100 μm; iii, 50 μm; iv, 25 μm. Data are presented as mean ± SD (n = 3). Multiple comparisons were analyzed using two-way ANOVA with Tukey's correction. ∗∗∗∗p < 0.0001.

Based on the novelty, and demonstrated efficiency and biocompatibility of the NL37 lipid, together with the well-documented systemic toxicity of lipofectamine *in vivo*, NL37 was selected as the carrier for BMP-7 cmRNA delivery in the subcutaneous implantation study. Physicochemical characterization of BMP-7 cmRNA/NL37 lipoplexes carrying the different investigated amounts of cmRNA is shown in Fig. 4a–c. Notably, the lipoplex size increased approximately 15-fold at the highest mRNA concentration (3.6 μg; p < 0.0001). This effect was independent of the mRNA sequence, as an equivalent size increase was observed when 3.6 μg of NC cmRNA was used, indicating that the change was driven by the total mRNA amount rather than its identity. Remarkably, the electrokinetic potential of all lipoplexes remained in the same (positive) range, independently of the cmRNA amount or identity. TEM imaging of the cmRNA/lipid particles revealed spherical structures with a characteristic core–shell morphology, consisting of an outer lipid layer surrounding a central core in which the mRNA is encapsulated (Fig. 4c). Further details on the obtained results can be found in the result section of the supplementary material.

To develop transcript-activated matrices (TAMs) to be implanted, BMP-7 cmRNA/NL37 lipoplexes were loaded into fibrin–CaP scaffolds. Fig. 4d and e shows morphological characteristics of the scaffolds. In Fig. 4d, stereomicroscope images of the front and side views illustrate the hydrogel's texture, showing well-dispersed CaP particles clearly visible throughout the scaffold. Scanning electron micrographs (Fig. 4e–i–iv) provide a detailed visualization of the scaffold surface. At lower magnification, the outer surface appears relatively smooth, with CaP granules embedded within the matrix (Fig. 4e–i). Higher magnification images reveal the dense, interwoven fibrillar network characteristic of fibrin scaffolds (Fig. 4e, ii–iv). Within this network, CaP granules are clearly identifiable as irregular, roughly spherical to angular particles integrated throughout the mesh. Their rough surface morphology and uniform distribution indicate effective incorporation into the scaffold matrix.

### 3.9 BMP-7 cmRNA remains local to the site of administration and results in successful BMP-7 protein production

**Fig. 5:**
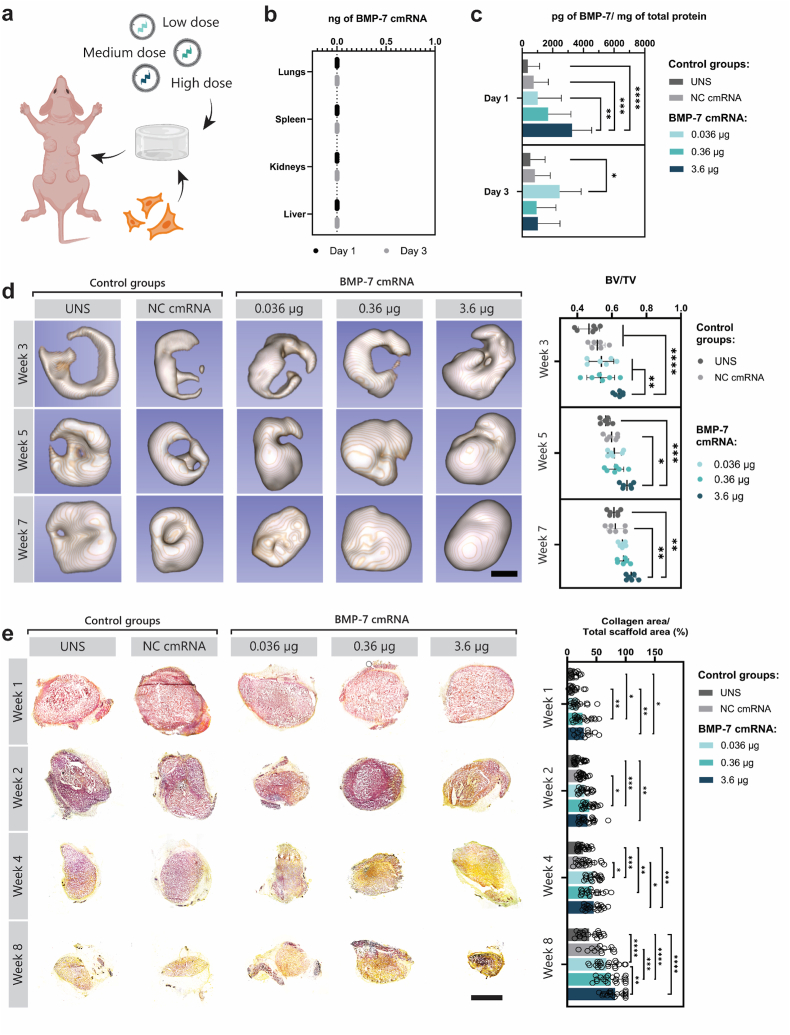
| cmRNA biodistribution, BMP-7 production, and kinetics of bone formation. (**a**) Schematic of loaded scaffolds used for subcutaneous implantation in nude mouse. (**b**) Absence of implanted cmRNA in collected organs. (**c**) Protein expression of BMP-7 after 1 or 3 days of implantation. (**d**) Representative images of 3D reconstructed μCT scans and their BV/TV quantification. (**e**) Panel with representative images of explants, the staining colors elastic fibers and nuclei black/blue, collagen yellow, mucin bright blue, fibrin bright red, and muscle red. Percentage of yellow-stained collagen areas obtained with post-imaging processing. Data are presented as mean ± SD (n = 9 for ELISAs measurements, n = 6 for μCT scans, n = 20 images/condition for movat pentachrome staining). Scale bar, 2 mm. Multiple comparisons were analyzed using two-way ANOVA with Tukey's correction and Sidak's correction for protein production and BV/TV, respectively. ∗p < 0.05, ∗∗p < 0.01, ∗∗∗p < 0.001, ∗∗∗∗p < 0.0001.

Fig. 5a schematics illustrate the approach followed in our study for the subcutaneous implantation of BMP-7 cmRNA/NL37 lipoplexes loaded TAMs in mice. Implantation of the TAMs showed no detectable cmRNA in the lungs, spleen, kidneys, or liver at days 1 and 3 post-administration (Fig. 5b), indicating localized retention at the implantation site. Local BMP-7 protein production was dose-dependent at day 1 (Fig. 5c), with the highest dose (i.e., 3.6 μg) exceeding the 3000 pg BMP-7 protein produced, which was significantly higher than untransfected and NC cmRNA controls (*p < *0.0001). Notably, the amount of BMP-7 produced by employing the highest dose of BMP-7 cmRNA was 3-fold higher than the amount produced when the lower cmRNA dose was used (day 1; *p = *0.0023). Surprisingly, by day 3, the lowest cmRNA dose (i.e., 0.036 μg) exhibited the highest BMP-7 levels (2458 ± 1394 pg/mg protein), although no statistical significance could be demonstrated between the different BMP-7 cmRNA doses investigated (*p* > 0.05).

### 3.10 BMP-7 cmRNA TAM boosts stem cells to deposit bone-like tissue *in vivo*

The formation of ossified tissue was evident in all implanted scaffolds (Fig. 5d). Notably, quantitative bone volume–to–tissue volume ratios (BV/TV), derived from μCT scans, showed significantly higher BV/TV at week 3 in the TAM group featuring the higher dose cmRNA (i.e., 3.6 μg of BMP-7 cmRNA) compared to controls (*p < *0.0001) and to the low and medium cmRNA dose TAM groups (*p < *0.0022). By weeks 5 and 7, no significant differences were observed among BMP-7 cmRNA TAM groups. Nevertheless, BV/TV values for the BMP-7 cmRNA TAM groups remained significantly higher than controls (both UNS and NC cmRNA). Notably, bone-like tissue formation initiated at the scaffold periphery and progressively filled the internal volume of the construct.

### 3.11 Histology confirms progressive bone formation and shows TAM degradation

Movat's pentachrome staining revealed dynamic tissue remodeling within the scaffolds. Initial fibrin (red) was replaced by cellular infiltration (purple) by week 2, and subsequently by collagen matrix (yellow), indicative of bone formation at later stages (Fig. 5e). Quantification of the yellow-stained area showed significant, time-dependent increases in the medium and high dose BMP-7 cmRNA TAM groups compared to UNS and NC cmRNA controls. Concurrently, the scaffold area diminished, suggesting gradual fibrin degradation as new bony matrix developed.

### 3.12 BMP-7 cmRNA TAM supports osteogenic gene expression *in vivo*

**Fig. 6:**
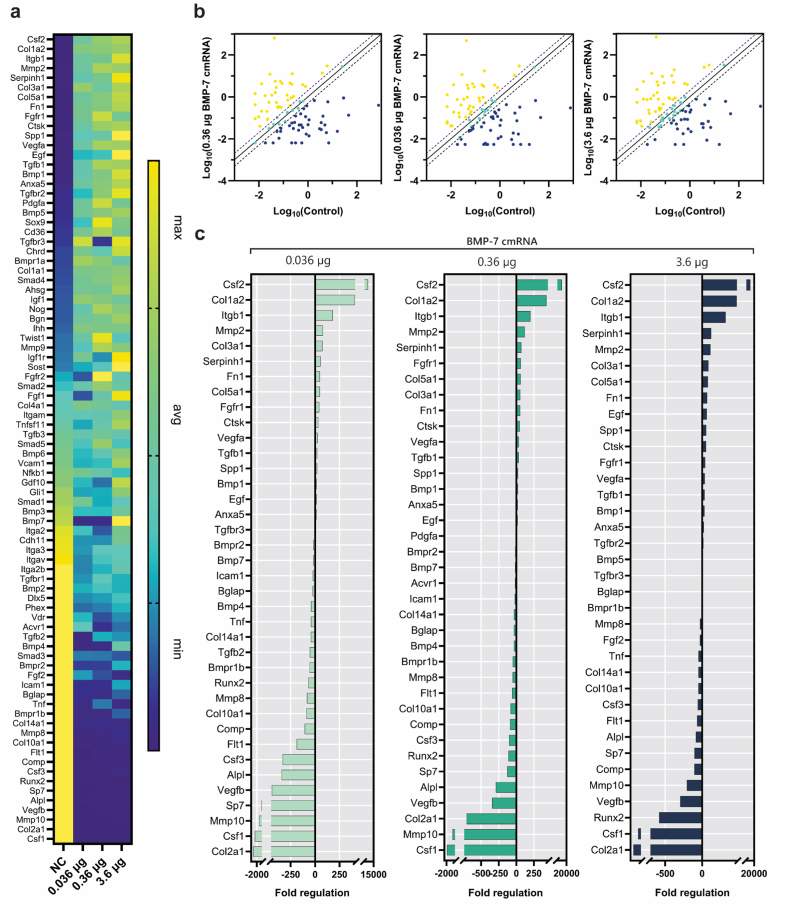
| Osteogenic gene expression in newly formed tissue after 1 week of implantation. (**a**) Heat maps illustrating the magnitude of gene expression of all osteogenic markers. (**b**) Scatter plots comparing normalized gene expression between treatment groups and the NC control group. (**c**) Bar graphs showing fold changes in gene upregulation and downregulation, calculated using the 2^−ΔΔCT^ method.

Expression of osteogenic genes at week 1 post-implantation revealed a BMP-7 mRNA-specific gene expression profile (Fig. 6a). The scatter plots (Fig. 6b) highlight the number of genes that are up- or downregulated by more than 2-fold in the BMP-7 cmRNA treatment groups compared to the NC cmRNA control group. Upregulated genes are depicted in yellow, unchanged genes in green, and downregulated genes in blue.

Among the three different BMP-7 cmRNA doses used, a distinct gene expression pattern could be observed (Fig. 6b and c), with the high-dose BMP-7 cmRNA showing a more pronounced upregulation (Fig. 6a). Specifically, osteogenic genes such as sclerostin (Sost), secreted phosphoprotein 1 (Spp1, also known as osteopontin), Bmp1, and Bmp7, growth factors Egf and Fgf1, collagen-related ECM protein Serpinh1, and adhesion gene integrin subunit beta 1 (Itgb1) were upregulated. Cell surface receptors Insulin-like Growth Factor 1 Receptor (Igf1r) and Transforming Growth Factor-beta Receptors (Tgfbr-2 and -3) also showed an upregulation in this group. Worth mentioning is the specific regulation of developmental transcription factors Sox9 and Twist1, as well as the growth factor Pdgfa, and receptors Fgfr-1 and -2 in the medium dose BMP-7 cmRNA group, while the low dose group showed upregulation of only Tgfbr-3, at similar levels to the high dose group.

**Fig. 7:**
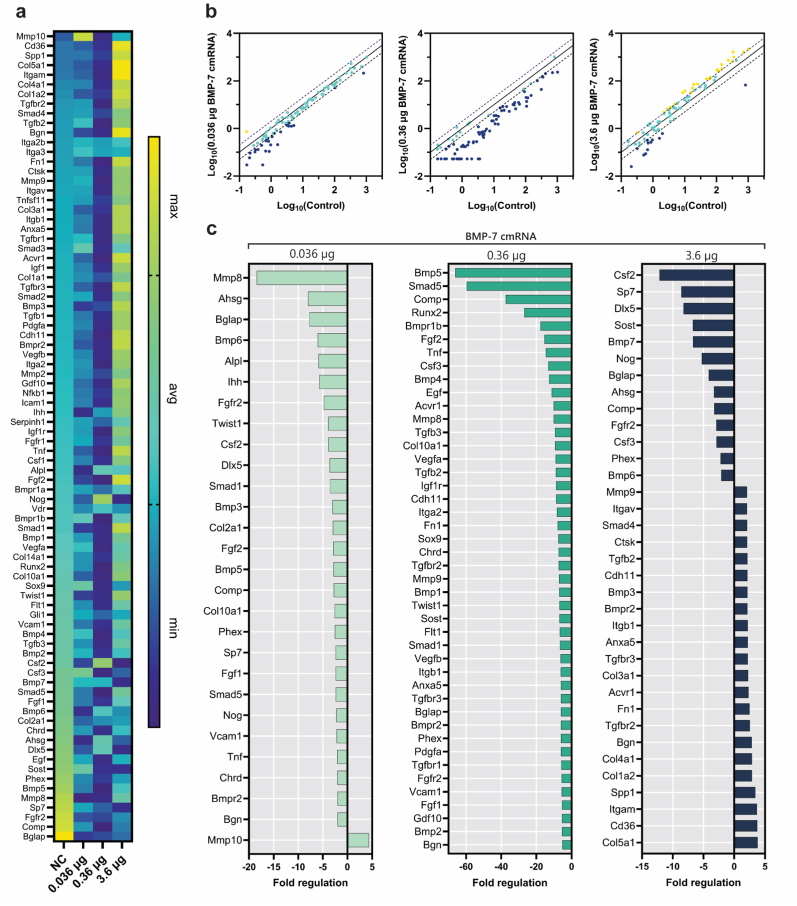
| Osteogenic gene expression in newly formed tissue after 8 weeks of implantation. (**a**) Heat maps illustrating the magnitude of gene expression of all osteogenic markers. (**b**) Scatter plots comparing normalized gene expression between treatment groups and the NC control group. (**c**) Bar graphs showing fold changes in gene upregulation and downregulation, calculated using the 2^−ΔΔCT^ method.

Gene expression was further investigated at weeks 2 (Fig. S17), 4 (Fig. S18), and 8 (Fig. 7) post-implantations. While at week 2, no notable gene expression was appreciated in any of the investigated groups, at week 4, the group administered a low dose of BMP-7 cmRNA showed specific upregulation of Bmpr2, osteogenic transcription factor Runx2, Col5a1, and Integrin alpha M (Itgam). Of note, the group receiving medium dose BMP-7 cmRNA uniquely showed high expression of Alpl at week 4 after implantation (>100-fold; Fig. S18a). Interestingly, at week 2 post-implantation, cartilage markers (Col2a1) and MMPs (Mmp8, Mmp9, Mmp10) were consistently downregulated across all the studied groups. Later, at week 4, a marked downregulation of genes was observed for the high dose BMP-7 cmRNA group with Runx2, Bmp4, and Tnf noticeably featuring a >15-fold downregulation (Fig. S18c).

At the latest observation time investigated, week 8 post-implantation, no distinguishable gene expression was recorded for the low and medium dose groups except for matrix metalloproteinase Mmp10 in the low dose BMP-7 cmRNA. (Fig. 7). Remarkably, expression of Spp1 and Itgb1 remained high for this time of observation in the high-dose BMP-7 cmRNA group. Among the highest upregulation of genes observed for this group at 8 weeks were genes Cd36 and Itgam. In addition, expression of collagens, i.e., Col5a1, Col1a2, Col4a1, and Col3a1, was also upregulated. Other ECM-relevant molecules, such as biglycan (Bgn) and fibronectin (Fn1), also showed an upregulation. BMPs-related genes, such as Bmpr2 and Bmp3, Tgfbr-2 and -3, Smad4, and Acvr1 were highly expressed; however, Bmp-6 and -7 were downregulated.

Notably, genes associated with angiogenesis (vascular cell adhesion molecule 1 (Vcam1), intercellular adhesion molecule 1 (Icam1), vascular endothelial growth factors-A and -B (Vegfa, Vegfb) were detected at various timepoints.

### 3.13 Immunostaining validates osteogenesis, vascularization, and innervation in BMP-7 cmRNA TAM

**Fig. 8:**
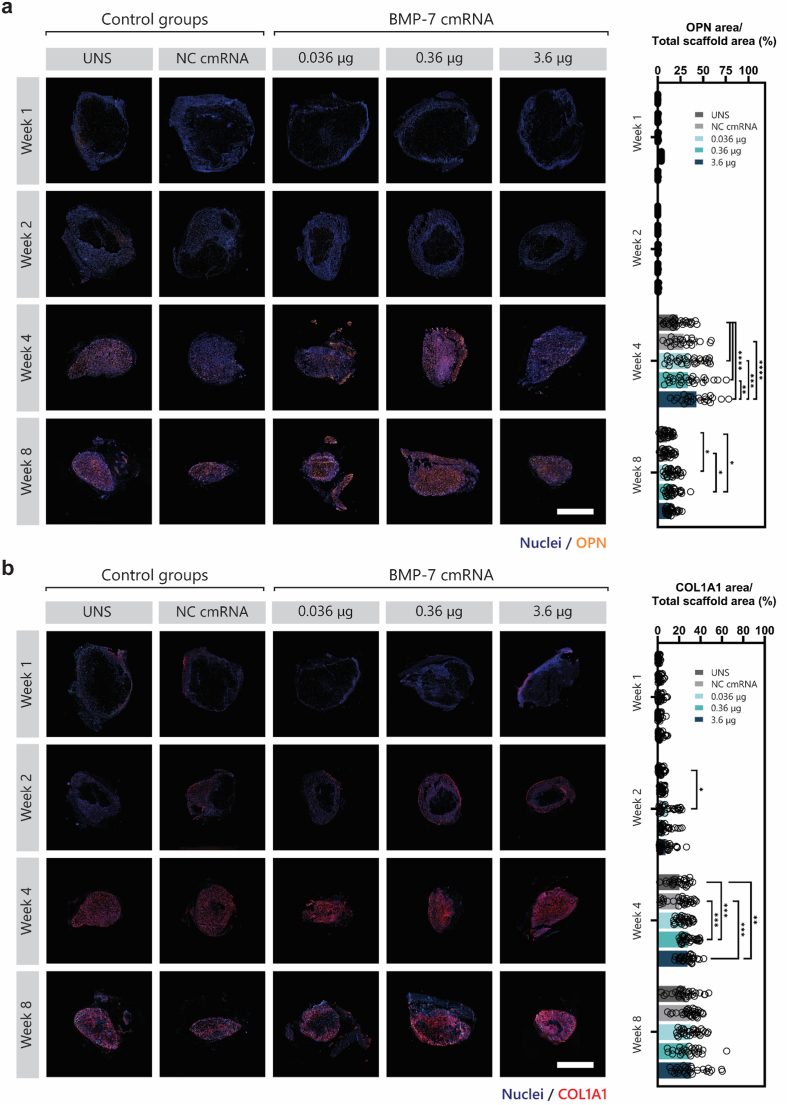
| Osteopontin and Type 1 Collagen Immunostainings of Explants. (**a**) Fluorescence microscopy images of scaffolds with DAPI (blue) for nuclear staining and osteopontin (OPN, orange) immunostaining. Quantification of OPN-positive areas through post-imaging analysis. (**b**) Fluorescence microscopy images showing DAPI (blue) for nuclei and type 1 collagen (COL1A1, red) immunostaining. Quantification of COL1A1-positive areas through post-imaging analysis. Data are expressed as mean ± SD (n = 30 images/condition). Scale bar: 2 mm. Statistical significance was determined using two-way ANOVA with Tukey's post-hoc test. ∗p < 0.05, ∗∗p < 0.01, ∗∗∗p < 0.001.

Osteopontin staining (orange, OPN; Fig. 8a) was negligible in early weeks but evident by week 4 across all groups. The staining quantification results revealed significantly higher OPN levels in groups receiving BMP-7 cmRNA TAMs compared to the UNS control (*p < *0.0005), with only the highest dose being markedly higher than the NC control (p < 0.0001). Collagen type I (red, COL1A1; Fig. 8b) presence was confirmed by immunofluorescence, primarily located at the periphery of the TAMs and became more prominent within by weeks 4 and 8 post-implantation, indicating increased collagen infiltration and bony-like tissue formation over time. Quantification of the COL1A1-positive area revealed an apparent increase across all BMP-7 cmRNA dose groups compared with the controls; however, only the low-dose group reached statistical significance at week 2 (*p* = 0.0101). By week 4, the medium and high-dose BMP-7 cmRNA groups were significantly higher than both, UNS and NC cmRNA controls. At week 8 post-cmRNA administration, no significant differences were observed across the investigated groups. Further evidence of osteogenesis within the BMP-7 cmRNA TAMs was assessed by evaluating areas positive for osteocalcin (magenta, OCN; Fig. S19) and F-actin filaments (shown in green; Fig. S19) at weeks 4 and 8 post-implantation. Cells identified by nuclear staining that co-expressed OCN and F-actin, highlighted by white arrows, were indicative of osteoblasts. A higher abundance of osteoblasts was observed surrounding the cavities within the TAMs, present across all groups. Additionally, what appears to be bone-lining cells, marked by white asterisks, were also identified.

**Fig. 9:**
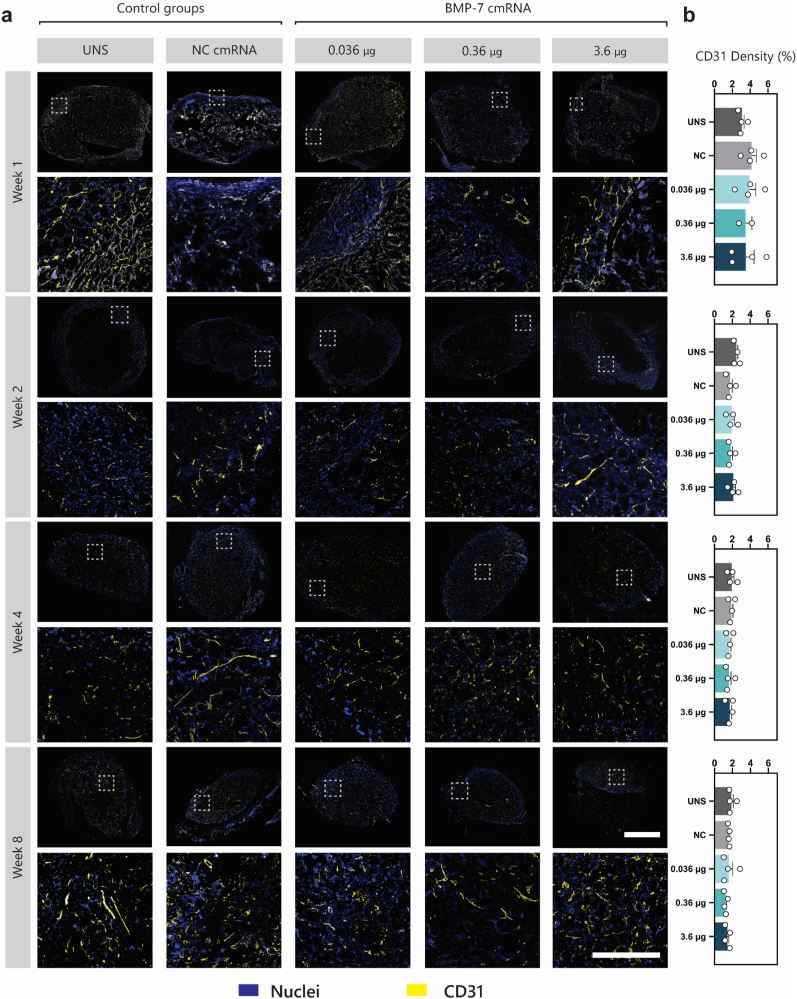
| Vascularization of Explants. (**a**) Representative fluorescence microscopy images of scaffolds stained with DAPI (blue) for nuclei and CD31 (yellow) for blood vessel identification. For each time point, the upper images display an overview of the entire scaffold area, while the lower images present a magnified view of specific regions. (**b**) Quantification of CD31-positive areas based on post-imaging analysis. Data are presented as mean ± SD (n = 4 images/condition). Scale bars: 2 mm (overview) and 100 μm (magnified view). A two-way ANOVA revealed no significant differences between groups.

CD31 staining, marking platelet endothelial cell adhesion molecule-1 (PECAM-1), revealed a greater abundance of blood vessels at early time points (yellow; Fig. 9a). Quantitative analysis showed that the CD31-positive area was highest at week 1 (approximately 4%) and declined to around 2% from week 2 onward (Fig. 7b). Early times of observation were investigated for vascular invasion, which showed an increase from 20 to 30% at week 1 to 40–70% by week 2 (Fig. S20). However, no statistical significance could be concluded on vascular invasion across the groups at any time of observation.

**Fig. 10:**
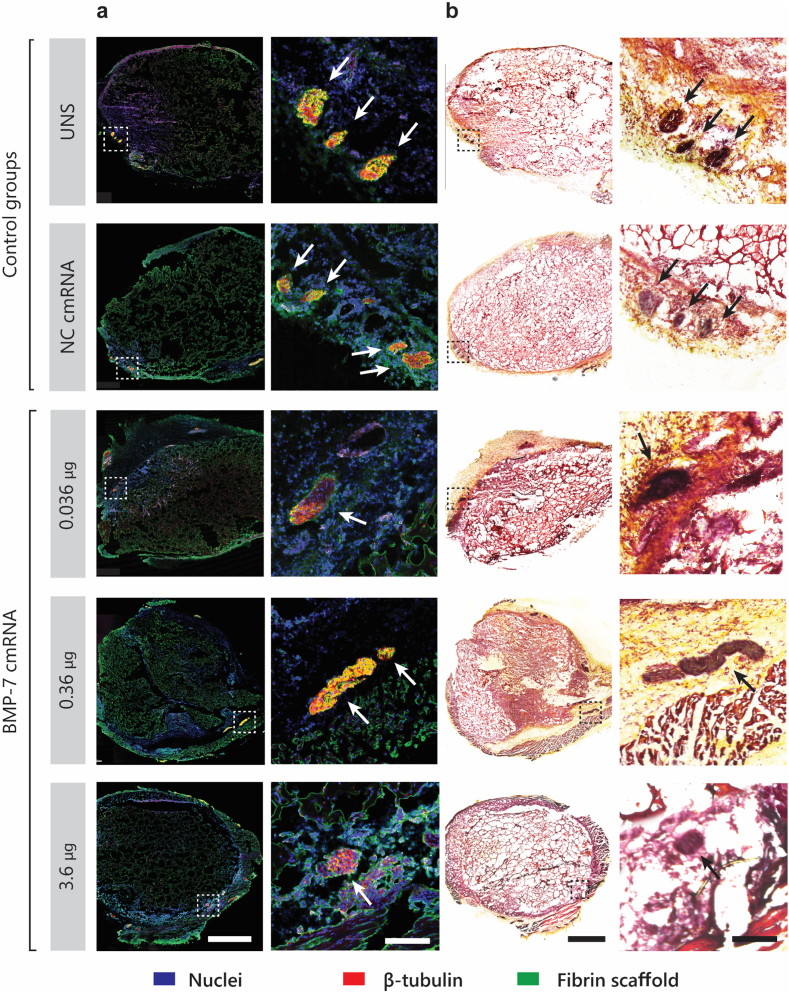
| Innervation of Explants at week 1 post-implantation. (**a**) Representative fluorescence microscopy images showing the entire scaffold area (left) and magnified regions (right). Nuclei are stained with DAPI (blue), while β-tubulin (red) highlights neuronal cells. Autofluorescence from the fibrin-CaP scaffolds is visible in green. (**b**) Representative brightfield microscopy images of the scaffolds, with Movat pentachrome staining. Regions of interest (ROIs) containing nerves are marked with dotted squares, and nerves are indicated by arrows. Scale bars: 1 mm (overview) and 500 μm (magnified view).

We consistently observed innervated tissue, as indicated by β3-tubulin staining (red, Fig. 10a) and the black or dark purple regions in the Movat pentachrome images (Fig. 10b), which represent stained elastic fibers. These fibers, composed of elastin and microfibrils, are typically found in all three connective layers of peripheral nerves. Innervation was most prominent in the peripheral regions of the implants. However, no clear differences in the extent of innervation were observed across groups or time points.

## 4 Discussion

Management of nonunions remains a major clinical challenge [11]. Substantial evidence indicates that BMPs can accelerate healing and reduce complications in severe nonunion cases, achieving outcomes comparable to autografts [10,44]. However, the widespread use of rhBMP-7 was constrained by the high costs associated with recombinant protein production. Whereas autologous graft procedures typically range from 338 to 1000 USD, commercially available sponges containing rhBMPs were priced at approximately 3500 USD [15]. Notably, rhBMP-7 is no longer available for clinical use. Dahabreh et al. analyzed the economic burden of tibial nonunion management and found that a substantial portion of the expenses associated with rhBMP-7 treatment stemmed from the biologic itself, with 41% of the total cost attributable solely to BMP production [12].

These economic constraints compound a fundamental biological limitation of BMP-based therapies: their reliance on supraphysiological doses to achieve osteoinductive effects. Such doses, often exceeding endogenous levels by more than a million-fold, not only drive up manufacturing costs but also contribute to safety concerns and variable clinical performance. Consequently, there is a pressing need for innovative, cost-effective, and safer BMP delivery systems capable of harnessing the osteogenic potential of BMPs and positioning them as a new gold standard for the treatment of challenging bone fractures.

In this work, lipid vectors integrated into biomaterial scaffolds were employed to deliver chemically modified BMP-7 mRNA *in vivo*, harnessing local cellular machinery for endogenous BMP-7 production and eliciting a pronounced osteogenic response. Chemically modified mRNAs encoding osteogenic factors, particularly BMP-2, have been developed for bone regeneration [[16], [17], [18],^33^,^45^]. In a recent study, it was demonstrated that co-delivery of BMP-2 and BMP-7 cmRNAs produced superior osteogenesis compared to either factor alone [45]. Motivated by these findings, we sought to determine whether BMP-7 cmRNA by itself could drive robust bone formation through complementary or parallel mechanisms. To address this question, we systematically evaluated BMP-7 cmRNA-mediated responses *in vitro* and *in vivo*. Notably, the *in vivo* osteogenic capacity of this construct had not been previously demonstrated. Importantly, this work extends beyond prior cmRNA-based osteogenic strategies by integrating systematic delivery vector optimization with multi-omics analysis, thereby linking delivery parameters to downstream biological responses.

Transfection with BMP-7 cmRNA supported robust osteogenesis *in vitro* and *in vivo*. The delivered cmRNA enabled endogenous BMP-7 protein production both in cultured cells and following *in vivo* administration, which in turn drove the observed osteogenic responses. *In vitro*, transfected hMSCs exhibited marked regulation of key osteogenic genes, consistent with BMP-7-mediated signaling activity, such as persistent upregulation of RUNX2 and the specific expression patterns for ALPL, BGLAP, and, at later times of observation, for SPP1. This is consistent with reported BMP signaling [46] and with previous observations for the dual BMP-2/-7 cmRNA delivery [45] and distinct from the BMP-2 cmRNA-induced genes [16,17]. *In vivo*, BMP-7 cmRNA provoked a marked ossification. In addition to the expected upregulation of diverse collagens and osteogenic markers, BMP-7 cmRNA activated cellular microtubule components. These are known to play a role in mechanotransduction [47,48], but also may be related to other processes such as cell migration [49]. Previous studies have described BMP-7's specific role in stabilizing microtubules [50], which supports our observations here. BMP-7 cmRNA also specifically activated pathways associated with axon guidance and VEGF/VEGFR2 signaling. Both processes are key regulators of innervation and angiogenesis, essential for bone healing, and typically precede mineralization *in vivo* [51,52]. Importantly, integrating transcriptomic and proteomic analyses with quantitative histological and immunostaining data provides cross-scale validation of the biological processes activated by BMP-7 cmRNA delivery. Rather than remaining at the level of pathway enrichment, the identified angiogenic, osteogenic, and neurogenic signatures are supported by functional tissue-level outcomes, linking molecular pathway activation to observable regenerative events *in vivo*. Additionally, PDGF signaling, a known stimulator of fibroblast and osteoblast growth, was also activated. *In vivo* ossification induced by BMP-7 cmRNA administration exhibited a clear dose-dependent response. This observation is consistent with previous findings for BMP-2 cmRNA, which demonstrated that doses exceeding 25 μg were required to achieve successful healing when administered orthotopically in a 5 mm critical-sized rat bone defect [17]. Together, these data support the notion that, at least for BMP-encoding cmRNAs, increasing the administered dose is linearly associated with enhanced osteogenic potential.

Notably, BMP-7 cmRNA administration resulted in strict local retention at the subcutaneous implantation site, with no detectable cmRNA in vital organs following implantation. This localized distribution is likely attributable to the use of transcript-activated matrices, or TAMs, as the delivery platform. Both key components of the TAM system, i.e., the lipid vector and the scaffold matrix, are expected to contribute to cmRNA retention and localized biological activity. In our study, lipid-based carriers were selected for mRNA delivery, as lipids remain among the most widely established and clinically advanced vectors for RNA therapeutics [53,54]. Our results indicate that key physicochemical parameters, including the lipid-to-nucleic acid ratio, backbone architecture (i.e., linear vs. branched), and overall charge, critically govern lipid vector performance. Branched lipids, for example, supported a wider range of effective mRNA-to-lipid ratios and cmRNA doses than their linear counterparts. This versatility is commonly attributed to their multivalent headgroups and additional hydrophobic chains, which enhance complex stability across varying formulation conditions and reduce aggregation or degradation [55,56]. Their branched architecture also presents a greater density of cationic groups capable of electrostatically engaging the anionic phosphate backbone of mRNA, thereby improving condensation and complex integrity [57]. In contrast, linear lipids depend on more rigid packing arrangements, limiting their ability to accommodate different mRNA-to-lipid ratios and resulting in narrower effective transfection windows. Remarkably, NL37, despite possessing a linear backbone, achieved the highest levels of BMP-7 production in hMSCs following BMP-7 cmRNA delivery. This performance is attributable to its high proportion of cationic lipid relative to co-lipids, which promotes efficient mRNA encapsulation through strong electrostatic interactions with the nucleic acid [58]. The resulting positively charged complexes exhibit enhanced affinity for the negatively charged cell membrane, facilitating cellular uptake, while the surplus cationic content aids endosomal escape by destabilizing endosomal membranes [59,60]. Together, these properties substantially increase transfection efficiency and protein yield. NL37 also demonstrated strong *in vitro* and *in vivo* biocompatibility, evidenced by the high metabolic activity of transfected cells (*in vitro*) and the absence of oxidative stress, heat shock protein induction, or activation of apoptotic pathways (*in vitro* and *in vivo*). This favorable profile aligns with literature reports describing comparable biocompatibility for linear lipid structures [61]. The development of an optimal matrix for RNA delivery remains an important yet incompletely resolved challenge in the field. In the present work, we employed fibrin-CaP hydrogels to explore a biomaterial composite well-known for its osteoconductive features [33,62], while employing two biomaterials of high biomedical value and clinically employed in tissue healing processes. The benefits of loading BMP-7 cmRNA to the fibrin-CaP hydrogel were evident by the early ossification, extensive bony-specific ECM production, and presence of osteoblast-like cells colonizing the gels.

Transcriptomic analysis of *in vivo* specimens revealed that TAM-mediated BMP-7 cmRNA delivery predominantly induced intramembranous ossification. This was evidenced by the significant upregulation of extracellular matrix components, including osteopontin (Spp1) and multiple collagen types (I, III, and V), in agreement with the histological and immunostaining findings. In contrast, chondrogenic markers such as collagen types II and X and Comp were strongly downregulated, and no cartilaginous tissue was detected. While previous studies have shown that rhBMP-7 can contribute to both intramembranous and endochondral ossification processes [63], earlier work using BMP-2 cmRNA in a large orthotopic segmental bone defect in rats demonstrated bone regeneration primarily via endochondral ossification [17]. These differences were anticipated and can be attributed to variations in the delivery route and the local microenvironment. In the present study, BMP-7 cmRNA was administered subcutaneously within an osteoconductive hydrogel containing human MSCs, creating a highly inductive and conducive niche to osteogenic differentiation. Under these conditions, the transplanted cells likely clustered and differentiated directly into osteoblast-like cells, serving as the initiating sites for intramembranous bone formation. In contrast, the orthotopic defect environment, characterized by a complex milieu of endogenous cells, signaling mediators, and extracellular matrix components, likely favored the endochondral ossification pathway observed following BMP-2 cmRNA delivery in previous study [17].

Varying timing of gene expression waves likely explains differences in bone formation based on BMP-7 dose. High doses triggered robust, immediate osteogenic activation, while lower doses led to a more gradual but sustained response, potentially due to reduced feedback inhibition [64]. Resurgence of osteogenic gene expression at week 8 with higher doses may indicate tissue maturation and recruitment of additional progenitor cells [65,66].

Bone tissue consists not only of a mineralized matrix but also of critical nervous and vascular components essential for healthy development. In our study, the BMP-7 cmRNA TAMs promoted both angiogenesis and innervation within the construct. The combination of BMP-7 cmRNA with the highly bioactive fibrin likely enhanced cell adhesion and migration, as fibrin is known to selectively recruit endothelial cells [67], Schwann cells [68], and neurons [69], all of which are expected to contribute to angiogenic and neurogenic processes. The porous structure provided by fibrin to the TAMs possibly facilitated cell infiltration and supported the formation of capillaries and nerve fibers, as previously reported for this biomaterial [70,71]. While no significant protein-level differences in angiogenesis or innervation were found, strong transcript expression of angiogenic markers (Vegfa, Vegfb, Mmp9, Col3a1) suggests interplay with BMP-7 cmRNA. Neural ingrowth was identified through β3-tubulin immunostaining and the presence of elastic fibers; however, these innervated regions were less prominent compared to vascularized areas. This observation highlights the potential of co-delivering cmRNA encoding neurotrophic factors, such as NGF, to further enhance both innervation and bone regeneration.

Collectively, our data demonstrate that BMP-7 cmRNA is a highly potent osteoinductive agent, capable of driving bone formation in environments lacking endogenous osteogenic cues. The subcutaneous implantation model employed here served as a proof of concept and enabled detailed characterization of essential product attributes, including *in vivo* functionality, biodistribution, and dose-response behavior. Nevertheless, further evaluation in clinically relevant fracture and nonunion models will be required to fully define the bone healing potential of BMP-7 cmRNA and support its translational advancement.

Importantly, this mRNA-based strategy represents a safe and controllable alternative to conventional gene and protein therapies, as it avoids permanent genomic modification while enabling transient, physiologically relevant BMP-7 production. By addressing long-standing economic and safety limitations associated with recombinant BMPs, this platform holds promise for transforming the treatment of difficult-to-heal bone fractures, including cases involving severe trauma, metabolic bone disorders such as osteoporosis, or fractures complicated by comorbidities. Given the large patient populations affected and the comparatively low manufacturing costs of cmRNA therapeutics relative to plasmid DNA, protein, or cell-based approaches [72], this technology offers a scalable and cost-effective pathway toward clinical implementation.

## Ethical approval

### 4.1 Animals

All experimental procedures complied with the guidelines of the Experiments on Animals Act (Wet op de Dierproeven, WOD). The project and its methods received approval from the Central Animal Testing Committee (Centrale Commissie Dierproeven, CCD) and Maastricht University's Animal Investigation Committee, under license number AVD10700202115299.

### 4.2 Cells

Human bone marrow mesenchymal stromal cells were isolated from bone marrow aspirates obtained from five healthy donors undergoing unrelated iliac crest surgeries. The collection and use of bone marrow samples were approved by the local ethical committee of the Maastricht University Medical Center, acting as the relevant Dutch authority, with the approval number METC 15-4-274. In addition, written informed consent was obtained from all donors in accordance with the Declaration of Helsinki.

## Data repository

Sequences of cmRNAs and primers used in our study can be found in the Dataverse repository:

https://doi.org/10.34894/LAJVWD.

## Ethics approval & consent to participate:

I testify, on behalf of all co-authors, that our submitted article under the title “**BMP-7 mRNA Delivered by Fibrin–CaP Scaffolds Activates Osteogenic Programs In Vivo as evidenced by transcriptomic and proteomic analyses**” followed ethical principles and was executed as approved by the relevant bodies. Corresponding information is provided below:

We confirm that any aspect of the work covered in this manuscript that has involved human patients or animal subjects have been conducted with the ethical approval of all relevant bodies and that such approvals are acknowledged within the manuscript.

List Ethics approvals:

License number (Animal experiment): AVD10700202115299
Approval received from:
Central Animal Testing Committee (Centrale Commissie Dierproeven, CCD) and Maastricht University's Animal Investigation Committee.
License number (Human material): METC 15-4-274
Specimen type: Bone marrow aspirates
Individuals: 5
Procedure: Iliac crest surgeries
Approval received from:
Local Ethical Committee of the Maastricht University Medical Center (MUMC+), acting as the relevant Dutch authority.
Written informed consent: Obtained from all donors in accordance with the Declaration of Helsinki.

## CRediT authorship contribution statement

**Claudia Del Toro Runzer:** Conceptualization, Formal analysis, Investigation, Methodology, Visualization, Writing – original draft, Writing – review & editing. **Nadia Roumans:** Investigation, Methodology. **Freek G. Bouwman:** Formal analysis, Investigation, Methodology, Resources. **Betzabeth Pereira Herrera:** Investigation, Methodology. **Berta Cillero-Pastor:** Investigation, Methodology. **Micaela Roque:** Investigation, Methodology. **Joëlle Amédée:** Funding acquisition, Writing – review & editing. **Elise Bovine:** Funding acquisition, Resources. **Florence Barrère de Groot:** Funding acquisition, Resources. **Christian Plank:** Funding acquisition, Resources. **Andrea Banfi:** Funding acquisition, Writing – review & editing. **Nunzia di Maggio:** Formal analysis, Investigation, Writing – review & editing. **Martijn van Griensven:** Conceptualization, Funding acquisition, Investigation, Methodology, Project administration, Supervision, Writing – review & editing. **Elizabeth R. Balmayor:** Conceptualization, Funding acquisition, Resources, Supervision, Visualization, Writing – original draft, Writing – review & editing.

## Declaration of competing interest

The authors declare the following financial interests/personal relationships which may be considered as potential competing interests.

Elizabeth R Balmayor reports financial support was provided by EU Framework Programme for Research and Innovation Future and Emerging Technologies. Martijn van Griensven reports financial support was provided by Horizon 2020 European Innovation Council Fast Track to Innovation. Christian Plank reports financial support was provided by Horizon 2020 European Innovation Council Fast Track to Innovation. Joelle Amedee reports financial support was provided by Horizon 2020 European Innovation Council Fast Track to Innovation. Elise Bovine reports financial support was provided by Horizon 2020 European Innovation Council Fast Track to Innovation. Florence Barrere-de Groot reports financial support was provided by Horizon 2020 European Innovation Council Fast Track to Innovation. Andrea Banfi reports financial support was provided by Horizon 2020 European Innovation Council Fast Track to Innovation. Elizabeth Balmayor has patent #PCT/US20180214572 issued to Inventor. Christian Plank has patent #PCT/US20180214572 issued to Inventor. If there are other authors, they declare that they have no known competing financial interests or personal relationships that could have appeared to influence the work reported in this paper.

## Data Availability

The main data supporting the results in this study are available within the paper and its Supplementary Information material. The structure and sequence of cmRNAs and primers used are available in the Dataverse repository ( https://doi.org/10.34894/LAJVWD). The raw and analyzed datasets generated during the study are available for research purposes from the corresponding author on reasonable request.
