# Supplementary material for: BMP-7 mRNA delivered by Fibrin–CaP scaffolds activates osteogenic programs in vivo as evidenced by transcriptomic and proteomic analyses
Source: Bioact Mater. 2026 May 29;65:107–27. doi: 10.1016/j.bioactmat.2026.05.046 (PMC13241941; doi:10.1016/j.bioactmat.2026.05.046)
Supplement: Multimedia component 1 [file mmc1.docx]

**Supplementary Information**

**BMP-7 mRNA Delivered by Fibrin–CaP Scaffolds Activates Osteogenic Programs In Vivo as evidenced by transcriptomic and proteomic analyses**

## **Supplementary Methods**

All chemicals, including the heat-inactivated Fetal Bovine Serum (FBS), were purchased from Sigma–Aldrich (St. Louis, MO, USA) unless otherwise stated.

### **Cells and cell culture**

Initial vector titrations were conducted using the immortalized human embryonic kidney 293 (HEK293) cell line (DSMZ, Leibniz, Germany). HEK293 cells were cultured in Dulbecco's Modified Eagle Medium (DMEM; Life Technologies, Carlsbad, CA, USA) supplemented with 10% heat-inactivated FBS (Sigma-Aldrich).

Subsequent experiments, including a narrower vector screening, proteomics analysis, osteogenic differentiation assays, and ectopic bone formation in mice through subcutaneous implantation, were performed using hMSCs at passages 3–5. These cells were isolated from bone marrow aspirates obtained from five healthy donors undergoing unrelated iliac crest surgeries. Detailed protocols for hMSC isolation, expansion, and characterization have been previously described ^83^. The collection and use of bone marrow samples were approved by the relevant ethical committees, acting as the relevant national authority. An ethical permit number will be provided here upon acceptance. In addition, written informed consent was obtained from all donors in accordance with the Declaration of Helsinki.

hMSCs were cultured in α-minimal essential medium (α-MEM; Life Technologies) supplemented with Glutamax, 10% heat-inactivated FBS, and 1% penicillin/streptomycin (P/S; 100 U/ml; Thermo Fisher Scientific, Waltham, MA, USA). Both HEK293 and hMSC cultures were maintained at 37°C in a humidified atmosphere containing 5% CO_2_.

For osteogenic differentiation assays, transfected hMSCs were cultured under osteogenic permissive conditions to evaluate the ability of BMP-7 cmRNAs to induce *in vitro* osteogenesis. The osteogenic permissive medium consisted of α-MEM supplemented with 2% FBS, 10 mM β-glycerophosphate, 200 µM ascorbic acid, and 1% P/S. Of note, dexamethasone was not added to this medium. Similarly, the osteogenic permissive medium was free of growth factors. Fresh medium was added every three days during the first week without removing the existing medium to maintain continuous exposure to secreted BMP-7. After the first week, a partial medium change was performed every three days, replacing half of the culture volume with freshly prepared osteogenic permissive medium.

### **Animals and ethical approval**

All experimental procedures complied with the guidelines of the Experiments on Animals Act. The project and its methods received approval from the Central Animal Testing Committee and the local University's Animal Investigation Committee. A license number will be provided here upon acceptance.

The study utilized a total of 88, eight-week-old female nude mice (BALB/cOlaHsd-Foxn1nu), weighing between 16 and 20 grams, sourced from a certified breeding facility (Envigo RMS B.V., Melderslo, the Netherlands). Before the experiment, the mice were acclimatized for two weeks in the university's Central Animal Facility under controlled conditions (24°C with a 12/12-hour light/dark cycle). Throughout the study, the mice were housed in groups of four per cage to support social interaction and well-being.

At each time point, five experimental groups were assessed: two control groups—scaffolds seeded with hMSCs only (UNS) and scaffolds with hMSCs plus non-coding cmRNA (NC)—and three treatment groups consisting of scaffolds with hMSCs loaded with low (0.036 µg), medium (0.36 µg), or high (3.6 µg) concentrations of BMP-7 cmRNA. Up to four scaffolds were randomly implanted per animal. For ELISA-based quantification of human BMP-7 production (performed on days 1 and 3), each condition included 9 replicates. For the remaining analyses, including µCT, PCR arrays, histology, and immunostaining, each condition was tested with 6 replicates.

**Lipids**

Overall, the vectors consist of patented lipid molecules (US9107931B2) with a modular structure comprising a hydrophobic unsaturated tail, a biocompatible linker, and a polar head group based on linear amino acid residues containing basic natural amino acids.

Each vector was formulated with co-lipids, including 1,2-dioleoyl-sn-glycero-3-phosphoethanolamine (DOPE), 1,2-dioleoyl-sn-glycero-3-phosphocholine (DOPC), and/or cholesterol. Formulation involved the thin-film hydration method, followed by sonication and extrusion through 0.2 µm and 0.1 µm polycarbonate membranes in 10 cycles. **Table S1** summarizes the key differences between the lipid vectors.

RmesFect™ and 3D-Fect™ shared a similar hydrophobic tail and biocompatible linker. Both are formulated using the same cationic lipid-to-co-lipid molar ratio. However, the polar head groups of the two vectors differed, with 3D-Fect™ presenting a branched backbone featuring 20% more positive charges compared to RmesFect™. In contrast, 3D-FectIN™, NL51, and NL37 belonged to a distinct class of lipids characterized by linear amino-acid-based polar heads, linked to a hydrophobic unsaturated tail, which are then dimerized using different bifunctional linkers of diverse geometries. These three lipids contained the same number of positive charges as RmesFect™ but exhibited differences in composition. For instance, 3D-FectIN™ and NL51 shared an identical co-lipid ratio, though NL51 had twice the overall lipid concentration. Meanwhile, NL37 was formulated with an excess of cationic lipid relative to co-lipids while maintaining a global lipid composition similar to that of 3D-FectIN™. Finally, Lipofectamine™ MessengerMAX™ (hereafter termed as LipoMM, Invitrogen, Waltham, MA, USA) consists of a mixture of 2,3‐dioleoyloxy‐N‐ [2(sperminecarboxamido)ethyl]‐N,N‐dimethyl‐1‐propaniminium trifluoroacetate) and DOPE.

**Characterization of BMP-7 cmRNA Lipoplexes**

BMP-7 cmRNA lipoplexes were prepared using the best-performing lipid vectors, NL37 (1:4 ratio) and LipoMM (1:1 ratio), as described before. For each condition, lipoplexes were formed in 800 µL of non-supplemented Opti-MEM containing 1 µg of BMP-7 cmRNA for *in vitro* experiments. Similarly, 800 μl of the lipid complexes with low (0.036 µg), medium (0.36 µg), or high (3.6 µg) BMP-7 cmRNA concentrations were used to characterize complexes used *in vivo*.

The size and zeta potential of the lipoplexes were measured using dynamic light scattering (DLS) on a Zetasizer Nano ZSP (Malvern Instruments, Worcestershire, UK). Measurements were performed at room temperature with a fixed angle of 173° backscattering. Electrophoretic mobility measurements were conducted using specialized cuvettes (DTS1070, Malvern Instruments). All samples were measured in triplicate (n = 3).

The morphology of the lipid complexes was examined by Transmission Electron Microscopy (TEM). A 5 μL aliquot of each lipoplex solution was applied to a 300-mesh copper grid coated with a carbon support film. The grids were air-dried overnight at room temperature before imaging. TEM analysis was performed using a Tecnai G2 Spirit BioTWIN iCorr microscope (FEI, Hillsboro, OR, USA) operated at 120 kV. Images were captured with a WA-Veleta camera (EMSIS, Münster, Germany).

### **cmRNA lipoplexes screening on reporter HEK293 cells**

Titration experiments were conducted to identify the lipid vector with the highest transfection efficiency and lowest cytotoxicity. For this, HEK293 cells were seeded in 96-well plates at a density of 80,000 cells/cm² and incubated for 24 hours prior to transfection. Cells were transfected with cmRNA encoding the reporter gene *Metridia Luciferase* (MetLuc). Information on the tested cmRNA-to-lipid ratios and cmRNA concentrations can be found in **Fig. S1**.

Lipoplexes were freshly prepared using non-supplemented Opti-MEM (Life Technologies). For each condition, the lipid vector was mixed with cmRNA at the highest tested concentration to form complexes, and subsequent dilutions were prepared serially. Immediately before transfection, the cell culture medium was removed, and 100 µL of the prepared lipoplexes were added to each well containing the cell monolayer. Untransfected cells served as negative controls.

At 6 hours of incubation of the complexes with the cells, the lipoplex-containing medium was replaced with fresh Opti-MEM supplemented with 10% FBS and 1% P/S. Supernatants were collected at days 1, 2, and 3 post-transfection for analysis.

### **Quantification of MetLuc Expression post-transfection in HEK293**

MetLuc expression was quantified by adding 50 µL of native coelenterazine (50 μM in degassed sodium phosphate buffer, pH 7.0; Synchem, Felsberg, Germany) to 50 µL of collected supernatant using white opaque 96-well plates. The occurring luminescent reaction is illustrated in **Fig. S1b**. Luminescence intensity was measured immediately at 480 nm using a CLARIOSTAR plate reader (BMG Labtech, Ortenberg, Germany) at room temperature. Results were reported as relative luminescence units (RLU). All measurements were conducted in triplicate (n = 3) across three independent plates for each lipid vector and time point.

### **Cytotoxicity Assessment post-transfection in HEK293**

Cytotoxicity of the lipid vectors was evaluated immediately following supernatant collection using the PrestoBlue assay (**Fig. S1c**), according to the manufacturer’s protocol (Thermo Fisher Scientific). Briefly, 10 µL of PrestoBlue reagent was added to 90 µL of non-supplemented Opti-MEM per well, and the cells were incubated for 1 hour under standard culture conditions. After incubation, the supernatants were transferred to a new 96-well plate for fluorescence measurements. Fluorescence was measured using a CLARIOSTAR plate reader (BMG Labtech) with an excitation wavelength of 535 nm and an emission wavelength of 615 nm.

Following fluorescence measurements, cells were washed three times with Dulbecco's Phosphate-Buffered Saline (DPBS), replenished with fresh Opti-MEM containing 10% FBS and 1% P/S, and returned to the incubator. Results were visualized using heatmaps, where higher relative fluorescence units (RFU, indicating higher metabolic activity) were depicted in green, and lower RFU (indicating lower metabolic activity) were depicted in red. All measurements were conducted in triplicate (n = 3) across three independent plates for each lipid vector and time point. To further correlate cell viability with metabolic assay, brightfield images Nikon Ti-S/L100 microscope (Nikon Europe, Amsterdam, the Netherlands) were acquired at selected wells to observe different levels of cytotoxicity induced by transfections (**Fig. S2**).

### **Preparation of scaffolds and transfection complexes for *in vivo* implantation**

Three different doses of BMP-7 cmRNA were tested, i.e., low (0.036 µg), medium (0.36 µg), and high (3.6 µg). Two control groups were included: an untransfected group without cmRNA, and a group with 3.6 µl of scrambled, non-coding NC cmRNA. Complexes were freshly prepared by combining the cmRNA with 14.4 µl of the NL37 lipid vector (maintaining a nucleic acid-to-vector ratio of 1:4), followed by a 15-minute incubation at room temperature.

For scaffold preparation, 30 µl of fibrinogen (Tisseel, Baxter, Deerfield, IL, USA) was combined with the prepared cmRNA complexes and 13.8 µl of Opti-MEM, resulting in a total volume of 60 µl (tube A). In a separate tube (tube B), 24 µl of CaCl₂ buffer (final concentration 20 mM) was mixed with 31.2 µl of Hepes buffer and 4.8 µl of thrombin (stock 500 U/ml, final concentration 20 U/ml). Meanwhile, 10 mg of calcium phosphate granules (granule size 45–106 µm, Kuros Biosciences, Bilthoven, the Netherlands) were presoaked in Opti-MEM in a non-treated 96-well plate, and excess Opti-MEM was removed before resuspension in 60 µl of the solution from tube B.

One million hMSCs were harvested and centrifuged at 500 x g for 5 minutes. The resulting cell pellets were resuspended in 60 µl of the solution from tube A, and this mixture was added to the wells containing the calcium phosphate granules. The contents were mixed by pipetting to ensure thorough homogenization. Plates were incubated under standard cell culture conditions (37°C, 5% CO₂) for 10 minutes. After incubation, the constructs were transferred to a new 6-well plate containing 5 ml of Opti-MEM and returned to standard culture conditions until further use.

### **Scaffold characterization**

The fabricated Fibrin-CaP scaffolds were first inspected macroscopically using a Nikon SMZ25 automated stereomicroscope, equipped with a 2x objective and PHOTONIC LED-Set Ringlight for enhanced visualization. To assess the microarchitecture, scanning electron microscopy (SEM) was employed. The scaffolds were dehydrated through a graded ethanol series (30%, 50%, 70%, 80%, 90%, 96%, and 100%), with each step lasting 30 minutes. Following dehydration, the ethanol was removed using a critical point dryer (EM CPD300, Leica, Wetzlar, Germany). The scaffolds were then coated with a thin layer of gold using a sputter coater (SC7620, Quorum Technologies, Lewes, UK). SEM imaging was performed with a JSM-IT200 (Jeol Ltd., Tokyo, Japan) at magnifications of 30×, 270×, 500×, and 1000×, under an accelerating voltage of 10 kV and a working distance of 10 mm.

## **Supplementary Results**

### **Selection of Lipid Vectors Based on Transfection Efficiency**

To identify the lipid vector with the highest transfection efficiency and lowest cytotoxicity, a series of titrations were conducted by transfecting HEK293 cells with *Metridia* Luciferase (MetLuc) cmRNA (**Fig. S5, Fig. S6**). Results are presented as heatmaps, providing a clear visualization of vector performance across different conditions.

Protein expression levels were quantified through luminescence measurements (**Fig. S5**) and are reported as relative light units (RLU). In the heatmaps, a darker blue color represents higher MetLuc protein production, while white indicates no detectable protein expression.

RmesFect™ resulted in high protein production for a broad range of cmRNA concentrations and cmRNA-to-lipid ratios, at both day 1 and day 2 post-transfection. Specifically, at day 2, high RLU levels were maintained for RNA-to-lipid ratios of 1:0.5 to 1:6, using cmRNA doses of 0.125 µg or higher. By day 3, however, expression levels decreased significantly, with only low RLU observed at a ratio of 1:2. In contrast, NL51 exhibited high protein production exclusively at day 1, with optimal performance at a ratio of 1:3 and cmRNA doses of 0.125 or 0.625 µg. NL37 supported elevated protein expression at higher cmRNA-to-lipid ratios (1:4 to 1:9), particularly with cmRNA doses of 0.125 or 0.625 µg. Notably, sustained protein expression was observed at day 2 with 0.125 µg of cmRNA at ratios of 1:4 and 1:5. On one hand, 3D-FectIN™ consistently showed low protein expression across all conditions and time points. On the other hand, with 3D-Fect™ moderate protein levels were observed at day 1 with cmRNA doses of 0.125 to 0.5 µg at ratios of 1:4 to 1:8. Interestingly, protein expression improved at day 2 using doses of 0.0625 to 1 µg of cmRNA across all ratios. Lipofectamine™ MessengerMAX™ (LipoMM) exhibited a narrow range of functional conditions, with high protein expression at day 1 at low ratios (1:0.5 or 1:1) and cmRNA doses of 0.25 to 1 µg. Protein levels declined by day 2, with only limited functionality under the same low-ratio conditions.

### **Metabolic activity**

Metabolic activity was evaluated as an indicator of cytotoxicity using the Presto Blue assay. Heatmaps display relative fluorescence units (RFU), where green denotes conditions with higher cell viability, and red highlights conditions associated with cytotoxic effects. The viability measurements confirmed that higher cmRNA-to-vector ratios, combined with high concentrations of cmRNA (i.e., >0.25 µg/well), exhibit significant cytotoxic effects (**Fig. S6**).

Interestingly, some vectors, such as RmesFect™ and NL37, demonstrated high metabolic activity under most conditions, except at cmRNA doses of 0.5 or 1 µg or when cmRNA-to-lipid ratios of 1:4 or higher were used. This high metabolic activity was sustained over time. In contrast, vectors like 3D-FectIN™ and 3D-Fect™ showed reduced metabolic activity at days 2 and 3 post-transfection compared to day 1, even at low cmRNA doses and low cmRNA-to-lipid ratios.

Notably, transfections with the commonly used LipoMM exhibited high cytotoxicity for a broad range of ratios investigated, starting at ratios of 1:2 and extending up to 1:10, with cmRNA doses ranging from 0.0625 to 1 µg.

The correlation between low metabolic activity and high cytotoxicity was further supported by brightfield microscopy observations under different transfection conditions (**Fig. S2**). Wells with high metabolic activity (green on heatmaps) displayed healthy, intact cell monolayers (**Fig. S2**, **panels 1–3**). In contrast, wells with low metabolic activity (red on heatmaps) exhibited large amounts of cell debris, apoptotic bodies, and detached cells (**Fig. S2**, **panels 4–5**).

### **Complex characterization for in vitro experiments**

Lipoplexes formed with NL37 and BMP-7 cmRNA exhibited a mean hydrated diameter of 797.6 ± 11.7 nm when prepared in Opti-MEM (**Fig. S7c**). Interestingly, when the same lipoplexes were formed in water, their size was drastically reduced, measuring 77.3 ± 1.77 nm—approximately 10 times smaller (*p <* 0.0001). This suggests significant interactions between the media components and the complexation process. Empty NL37 vectors showed consistent sizes of 104.3 ± 16 nm in water and 190.5 ± 32.2 nm in Opti-MEM.

In comparison, LipoMM complexes exhibited greater consistency across conditions. The only statistically significant difference observed was between BMP-7 cmRNA lipoplexes formed with LipoMM in Opti-MEM, which had a mean size of 237.5 ± 24.5 nm, and the empty LipoMM complexes in the same medium, measuring 94.2 ± 13.9 nm (*p =* 0.0067).

Electrokinetic potential (z-potential) measurements (**Fig. S7d**) revealed significant differences based on the complexation medium. For NL37, z-potential values dropped significantly in Opti-MEM compared to water for both empty and BMP-7 cmRNA-loaded lipoplexes (*p <* 0.0001). Specifically, NL37 complexes in water exhibited a z-potential of 58.6 ± 11.4 mV, whereas in Opti-MEM, it decreased to 11.7 ± 0.49 mV, with no significant difference between loaded and empty vectors.

Conversely, LipoMM displayed negative z-potentials under all conditions. LipoMM (empty) ranged from -4 to -14 mV, while BMP-7 cmRNA-loaded complexes showed stronger negative potentials: -28.2 ± 4.3 mV in water and -26.3 ± 1.1 mV in Opti-MEM.

Transmission electron microscopy (TEM) analysis provided insight into the morphological features of BMP-7 cmRNA complexes (**Fig. S7e**). Both NL37 and LipoMM lipoplexes appeared as quasi-spherical particles, with LipoMM complexes exhibiting a smaller overall size. Both vector types showed the presence of inner vesicles. However, NL37 lipoplexes contained larger vesicles predominantly in the central region, while LipoMM lipoplexes featured smaller central vesicles and moderately larger vesicles toward the periphery.

### **Complex characterization for in vivo experiments**

Lipid complexes loaded with low and medium doses of BMP-7 cmRNA (0.036 µg and 0.36 µg) had sizes of 102.5 ± 10.3 nm and 127.2 ± 3.6 nm, respectively, which were significantly smaller than the high-dose BMP-7 cmRNA and non-coding (NC) cmRNA complexes (3.6 µg), with sizes of 1455 ± 35.2 nm and 1484 ± 153.5 nm, respectively (*p <* 0.0001) (**Fig. 4a**). In terms of charge, no significant differences were observed across the complexes, regardless of the cmRNA concentration, with z-potential values ranging between 13.3 and 15.5 mV (**Fig. 4b**).

Transmission electron microscopy also revealed a significant size difference between lipid complexes loaded with low and medium doses of BMP-7 cmRNA compared to those loaded with high concentrations of cmRNA (BMP-7 and NC control). Additionally, spherical particles with a distinct multivesicular structure were observed (**Fig. 4c**).

**Supplementary Tables**

**Table S1** | Summary of properties of the lipid vectors used for cellular internalization of cmRNAs.

**Table S2** | Gene list for the Osteogenic (PAMM-026Z) Qiagen PCR arrays used in the study (supplementary excel file).

**Table S3** | Proteomics of BMP-7 cmRNA-transfected hMSCs: Up- and downregulated proteins, including UniProt accession codes, abundance ratios (log2), and p-values (supplementary excel file).

**Supplementary Figures – Materials and Methods**


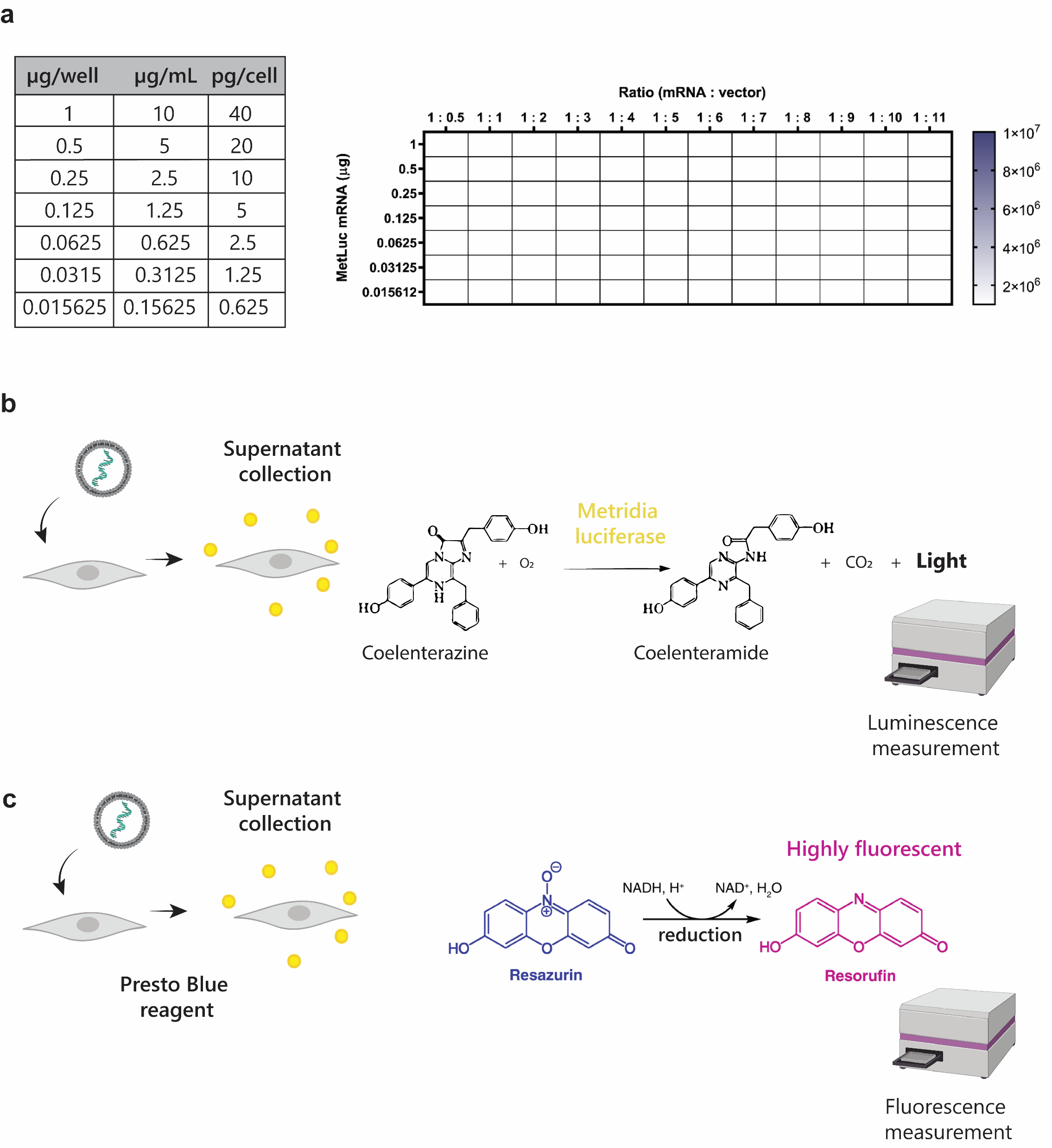


**Fig. S1 | Screening Setup.** (**a**) The table presents the concentrations of MetLuc cmRNA used for screening, expressed in µg/well, µg/mL, and pg/seeded cell. The layout illustrates the screening setup for multiple lipid vectors, where each row corresponds to different cmRNA concentrations, and each column represents varying cmRNA-to-vector ratios. (**b**) Schematic representation of the luminescence measurement principle, based on the reaction between Metridia luciferase and coelenterazine. (**c**) Illustration of the principle behind the Presto Blue assay, used to evaluate cellular metabolic activity.


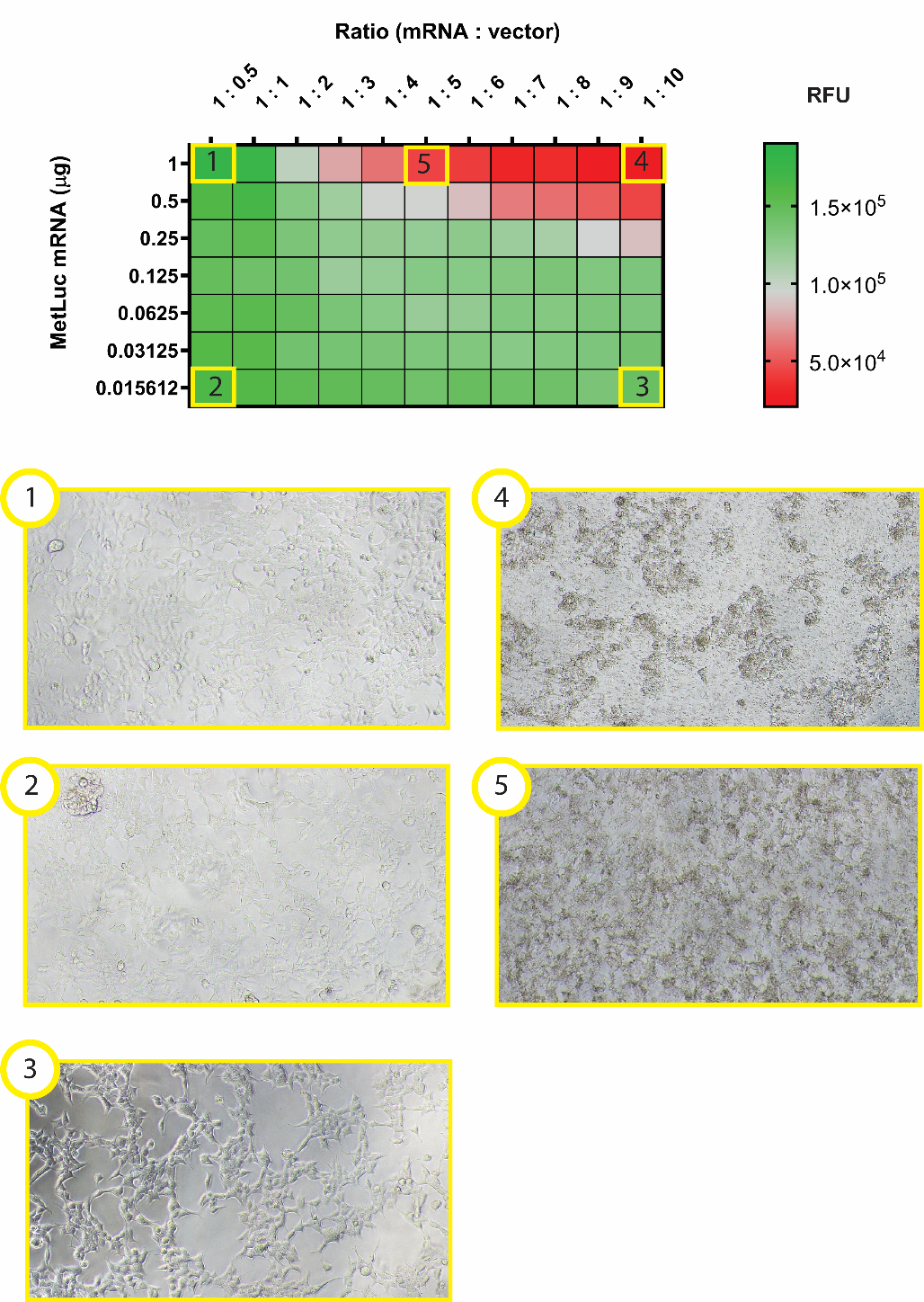


**Fig. S2 | Cytotoxicity of lipid vectors.** Representative heat map of cellular metabolic activity in transfected HEK293 cells, paired with brightfield images of corresponding wells, illustrating healthy cells (1–3) and apoptotic cells (4–5) post-transfection. Abbreviations: MetLuc, Metridia luciferase; RFU, relative fluorescent units.


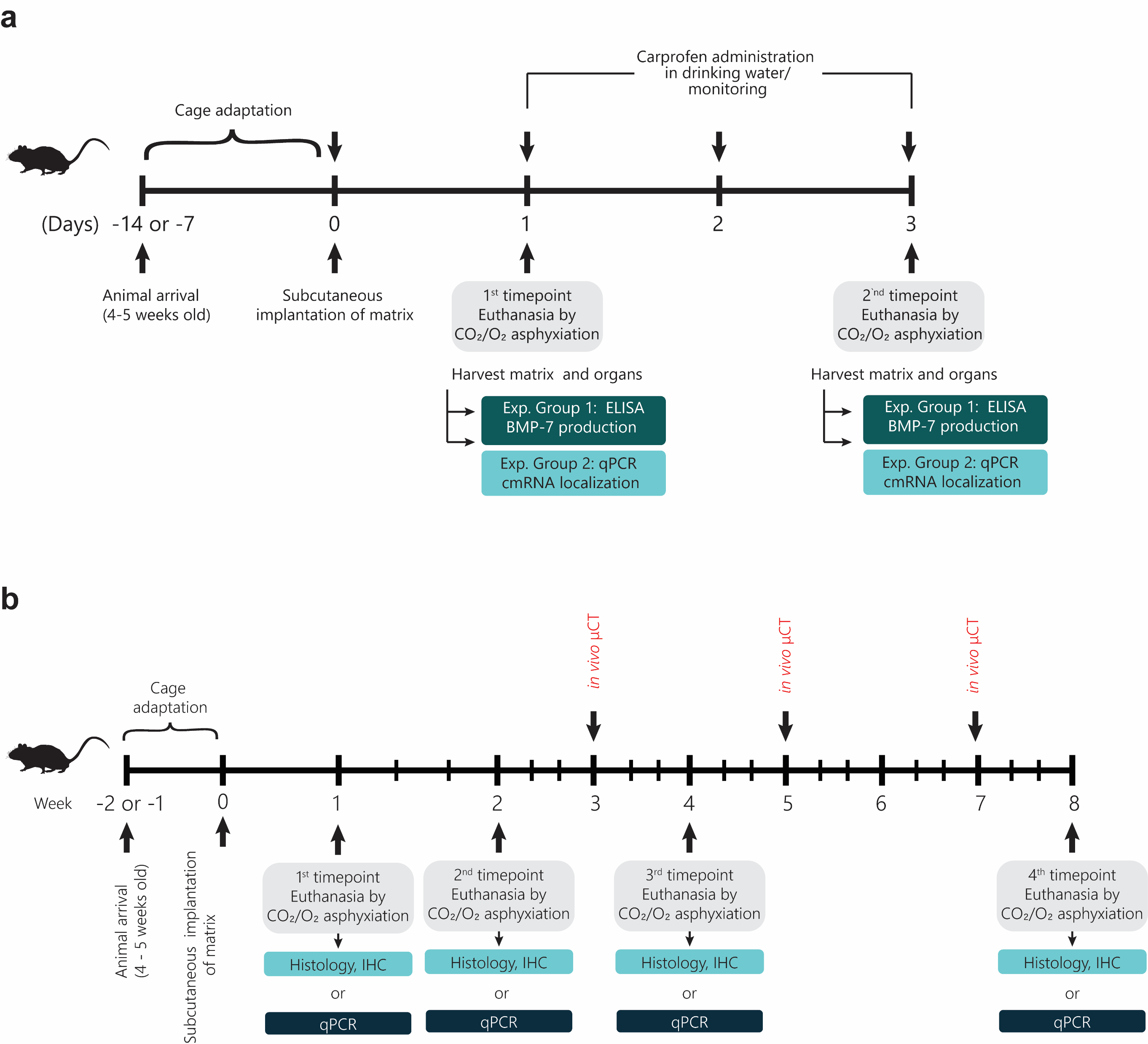


**Fig. S3 | Experimental Timelines for Subcutaneous Implantation in Mice and Subsequent Organ and Explant Collection.** (**a**) Timeline of phase 1 procedures, including scaffold collection for BMP-7 level assessment and organ collection to determine cmRNA localization. (**b**) Timeline indicating the time points for in vivo µCT scans, as well as explant collection for histology, immunostaining, and gene expression analysis.


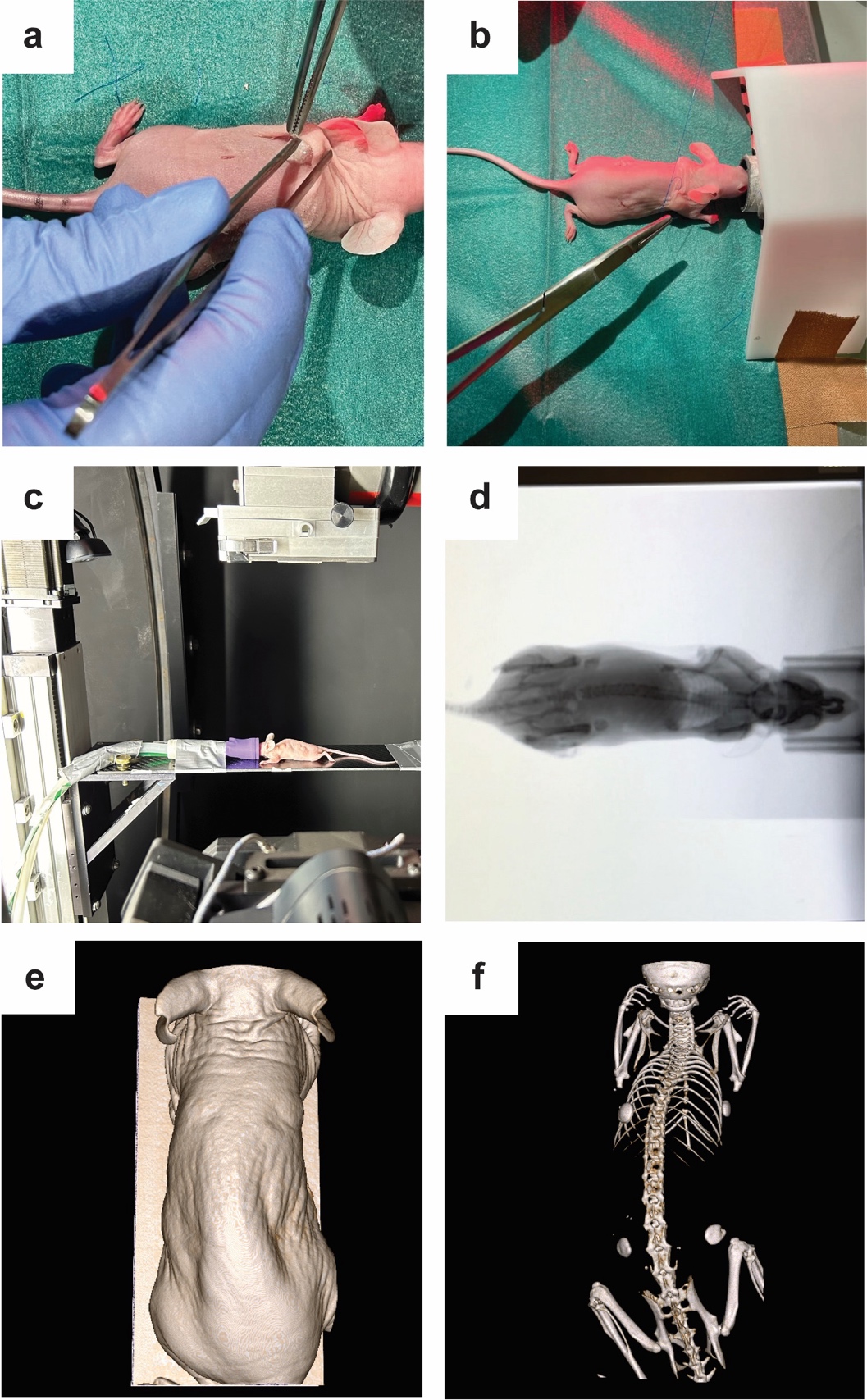


**Fig. S4 | Implantation of Fibrin-CaP Scaffold Loaded with BMP-7 cmRNA in Nude Mice and *In Vivo* µCT scans for ectopic bone formation.** (A) Implantation procedure: Isoflurane anesthesia was induced and maintained, followed by a 4 mm incision along the back, parallel to the spine. Subcutaneous pockets were created using surgical scissors, and four scaffolds were implanted per animal. (B) Incisions were closed with monofilament sutures. (C) µCT scans were performed using a small animal micro-irradiator, with anesthesia maintained via a nose cone inside the irradiator. (D) Transmission images were captured during a 360° rotational scan. (E & F) Mineralized scaffolds were visualized using 3D Slicer software, and bone volume was quantified through threshold-based segmentation and 3D measurement analyses.

**Supplementary Figures – Results**


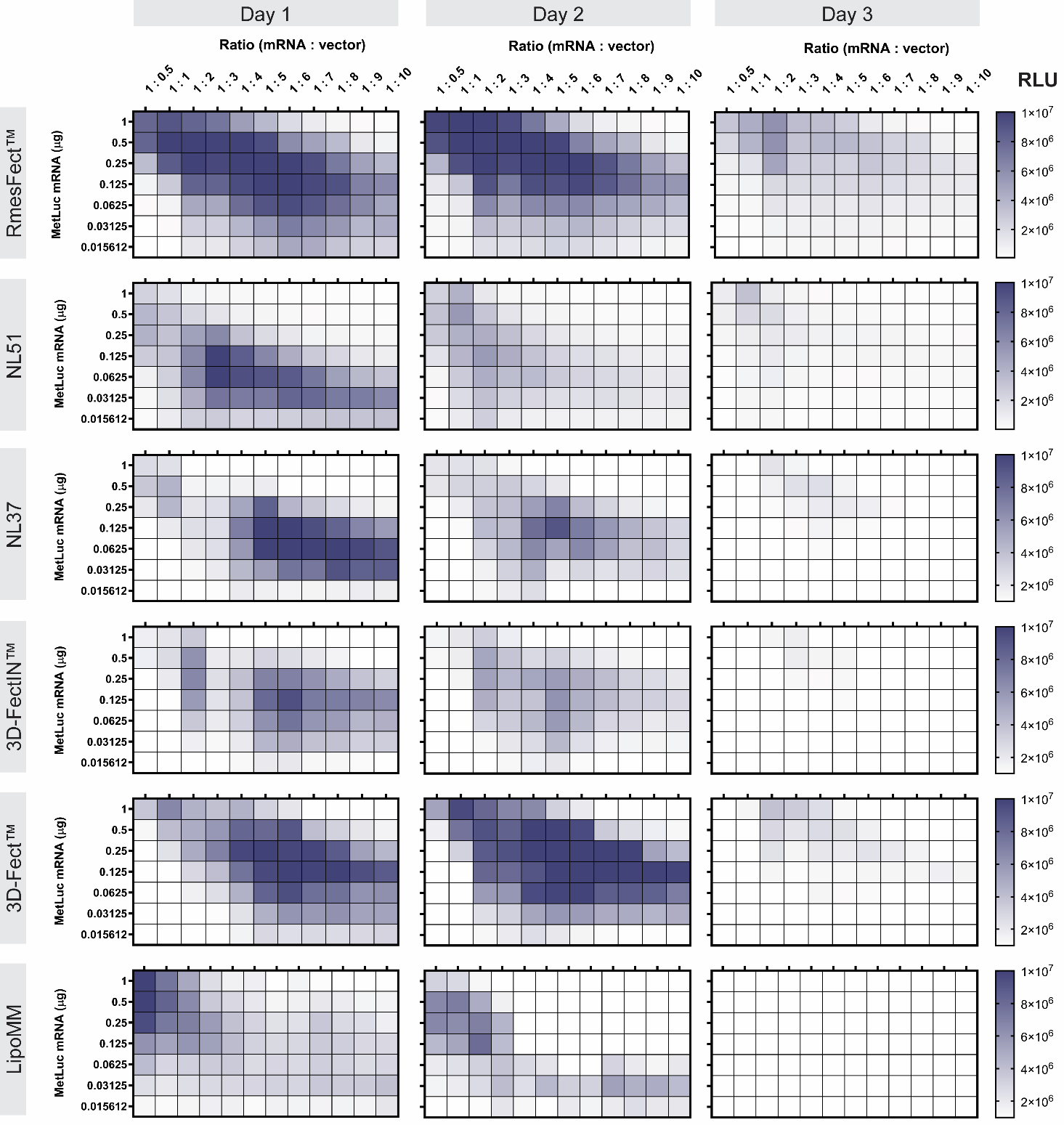


**Fig. S5 | Lipid vector screening for protein production efficiency.** Heat maps display protein production levels in HEK293 cells transfected with lipid complexes formed with *Metridia luciferase* cmRNA. Various cmRNA concentrations and cmRNA-to-lipid ratios were analyzed. Luminescence measurements were conducted on supernatants collected over 3 days post-transfection. Data represent the mean of three independent experiments. Abbreviations: MetLuc, *Metridia luciferase*; Lipofectamine™ MessengerMAX™, LipoMM; RLU, relative light units.


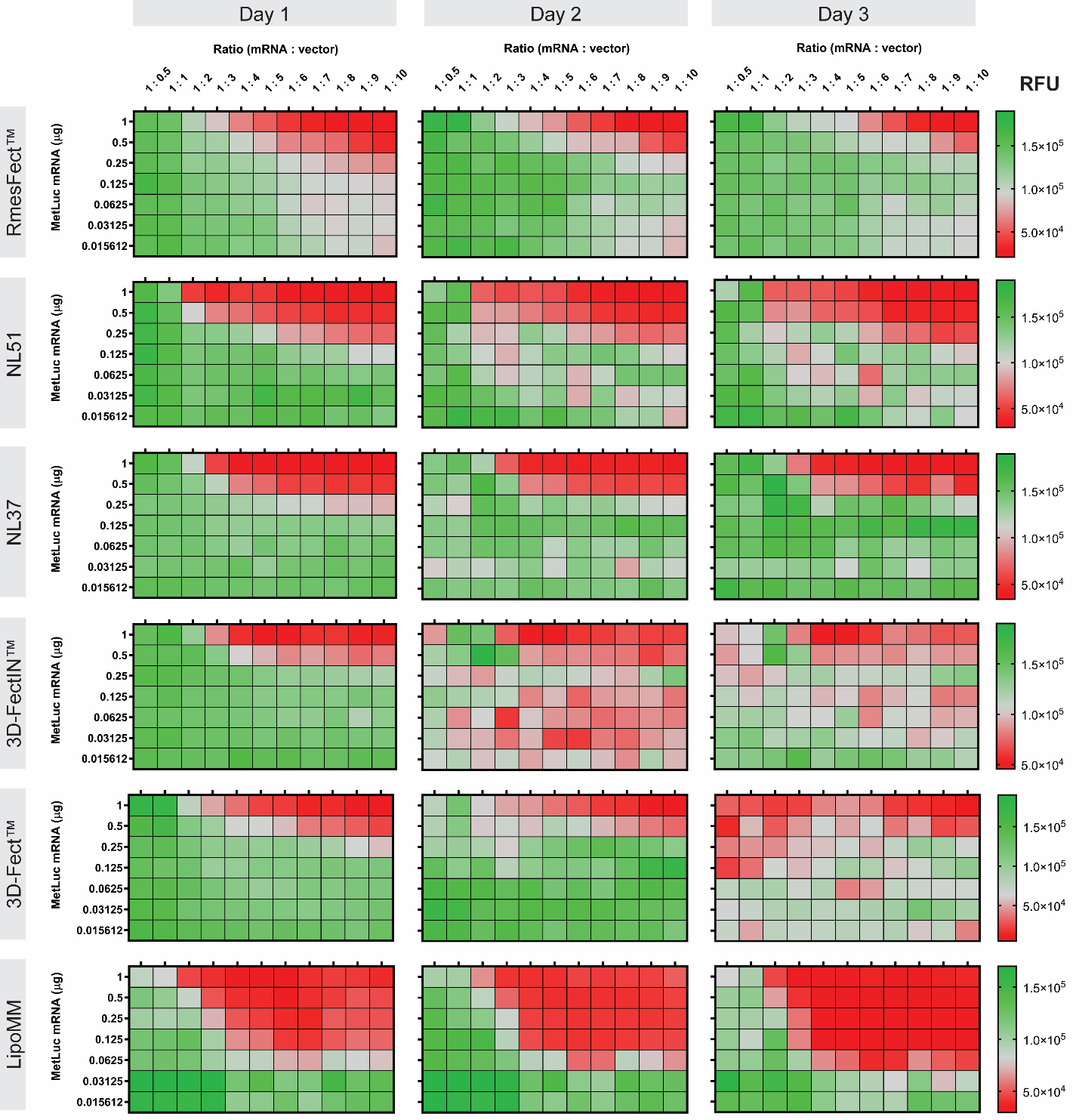


**Fig. S6 | Cytotoxicity assessment of lipid vectors.** Heat maps indicate cellular metabolic activity in HEK293 cells transfected with lipid complexes formed with *Metridia luciferase* cmRNA. Metabolic activity was evaluated using the Presto Blue assay, with fluorescence measurements performed on supernatants collected over 3 days post-transfection. Data represent the mean of three independent experiments. Abbreviations: MetLuc, *Metridia luciferase*; Lipofectamine™ MessengerMAX™, LipoMM; RFU, relative fluorescence units.


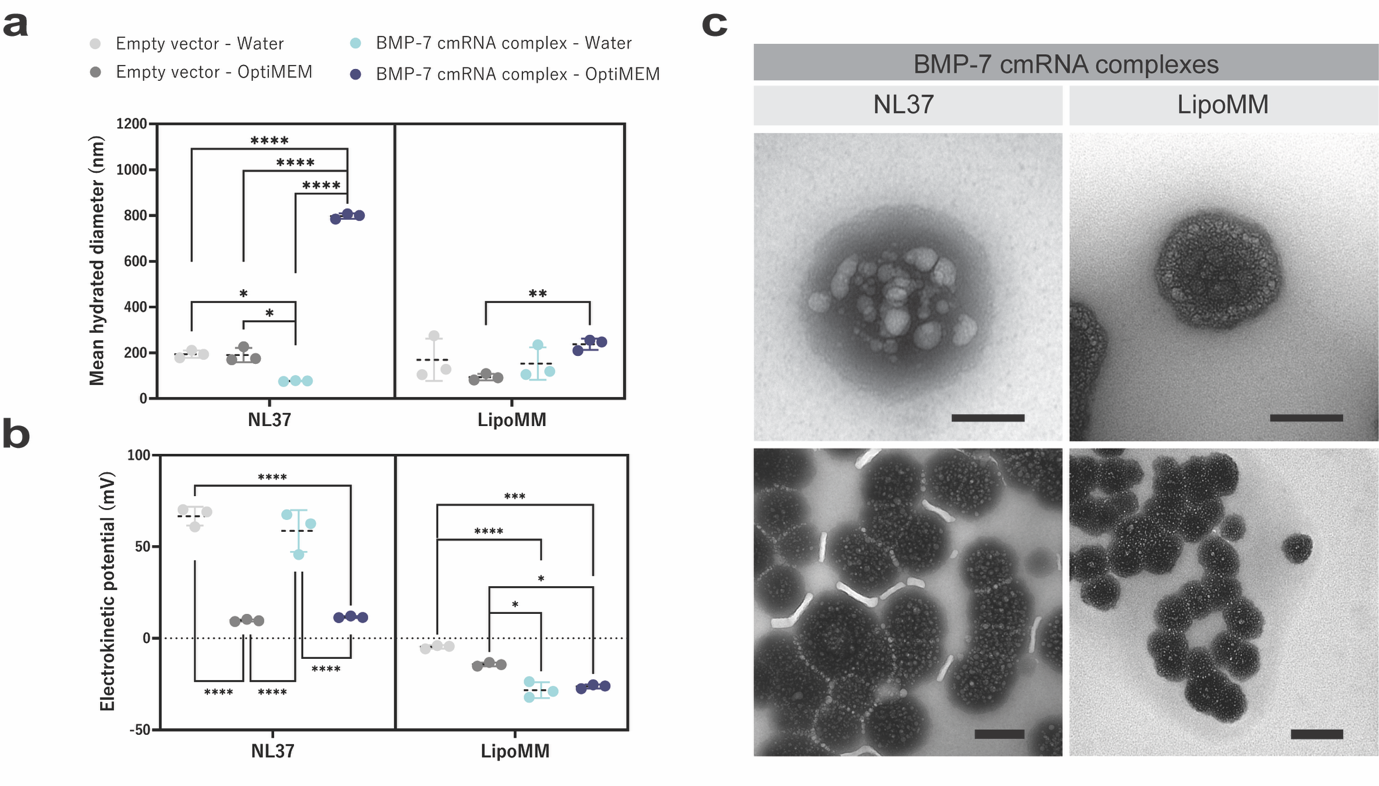


**Fig. S7 | Physicochemical characterization of BMP-7 cmRNA complexes with NL37 and LipoMM.** (**a**) Mean hydrated diameter, (**b**) electrokinetic potential, and (**c**) transmission electron microscopy photomicrographs depicting morphological features of the lipid complexes. Scale bars: 100 nm. Data are presented as mean ± SD (n = 3). Multiple comparisons were analyzed using two-way ANOVA with Šídák’s correction. *p < 0.05, **p < 0.01, ***p < 0.001, ****p < 0.0001.

**Fig. S8 | Panels showing ALP staining of hMSCs upon transfection with BMP-7 cmRNA.** Representative images of ALP staining at days 7, 14, 21, 28, and 35 post-transfections for donor 1 (**a**) and donor 2 (**b**). Scale bars: 500 µm.

**Fig. S9 | Panels showing ALP staining of hMSCs upon transfection with BMP-7 cmRNA.** Representative images of ALP staining at days 7, 14, 21, 28, and 35 post-transfections for donor 3 (**a**) and donor 4 (**b**). Scale bars: 500 µm

**Fig. S10 | Panels showing Alizarin red S staining of hMSCs upon transfection with BMP-7 cmRNA.** Representative images of Alizarin red S staining at days 21, 28, 35, 42, and 49 post-transfections of donor 1 (**a**) and donor 2 (**b**). Scale bars: 500 µm.

**Fig. S11 | Panels showing Alizarin red S staining of hMSCs upon transfection with BMP-7 cmRNA.** Representative images of Alizarin red S staining at days 21, 28, 35, 42, and 49 post-transfections of donor 3 (**a**) and donor 4 (**b**). Scale bars: 500 µm

**Fig. S12 | Panels showing osteopontin staining of hMSCs upon transfection with BMP-7 cmRNA.** Representative images of osteopontin stainings at days 21, 28, 35, 42, and 49 post-transfections of donor 1 (**a**) and donor 2 (**b**). Green = Osteopontin. Blue = DAPI (nuclei staining). Scale bars: 50 µm.

**Fig. S13 | Panels showing osteopontin staining of hMSCs upon transfection with BMP-7 cmRNA.** Representative images of osteopontin stainings at days 21, 28, 35, 42, and 49 post-transfections of donor 3 (**a**) and donor 4 (**b**). Green = Osteopontin. Blue = DAPI (nuclei staining). Scale bars: 50 µm.


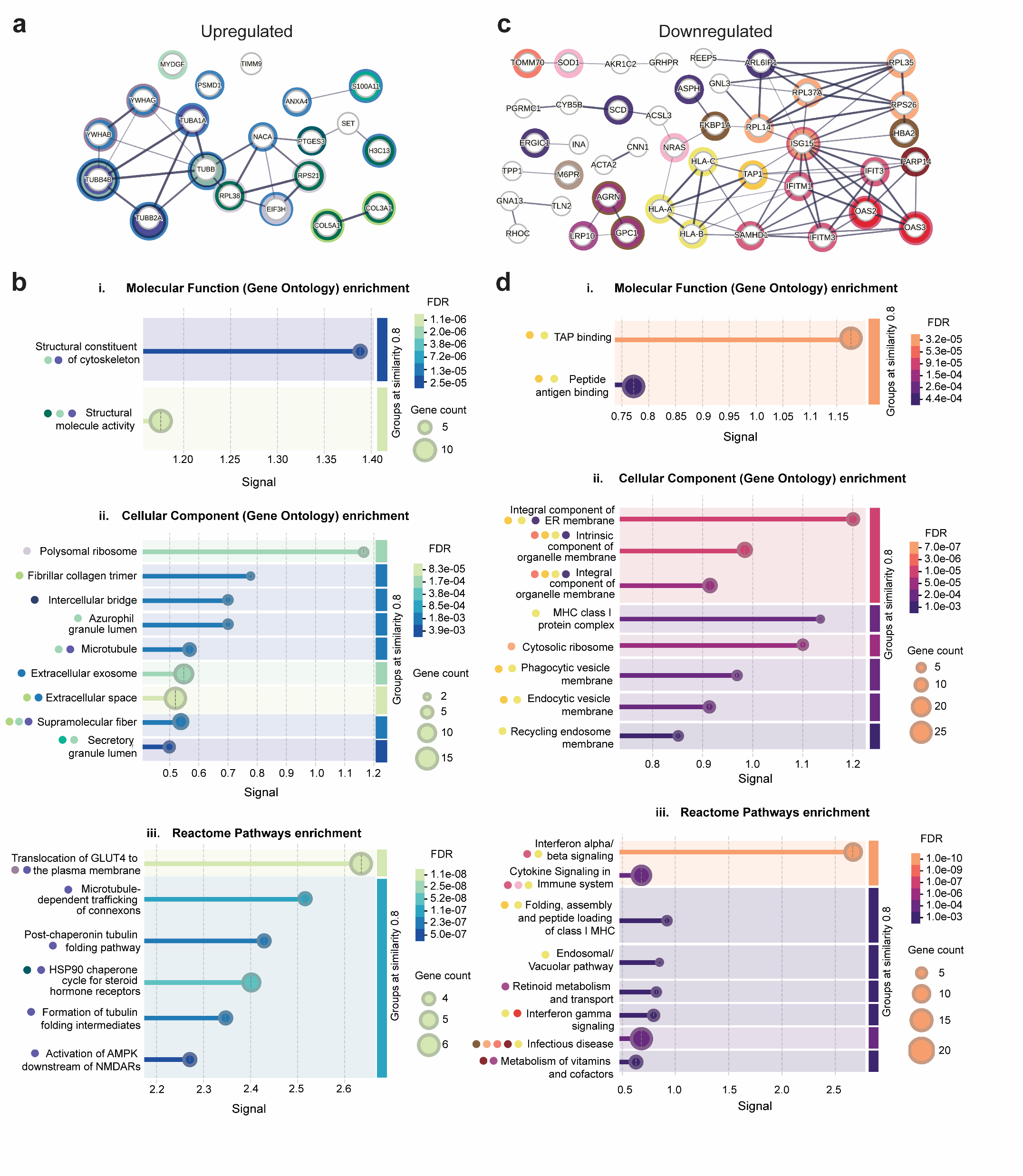


**Fig. S14 | Main biological changes induced by BMP-7 cmRNA transfections using the NL37 lipid vector.** (**a**) Protein interaction networks generated from proteomic analysis of BMP-7 cmRNA-transfected hMSCs, with non-coding cmRNA-transfected hMSCs as controls. Line thickness represents the strength of data support, and color of circles around each node indicate enrichment categories. (**i**) Network for upregulated proteins. (**ii**) Network for downregulated proteins. (**b** and **c**) Graphs illustrating key biological changes, including: (**i**) molecular functions, (**ii**) cellular components, and (**iii**) Reactome pathways. The y-axis represents enriched terms, while the x-axis shows their signal (a metric combining the observed/expected ratio and -log(FDR)). Line colors represent the FDR scale, and circle size indicates gene count. Color of circles at the left of each term relate to protein network in (a). Protein-protein interactions and enrichments were conducted using the STRING tool.


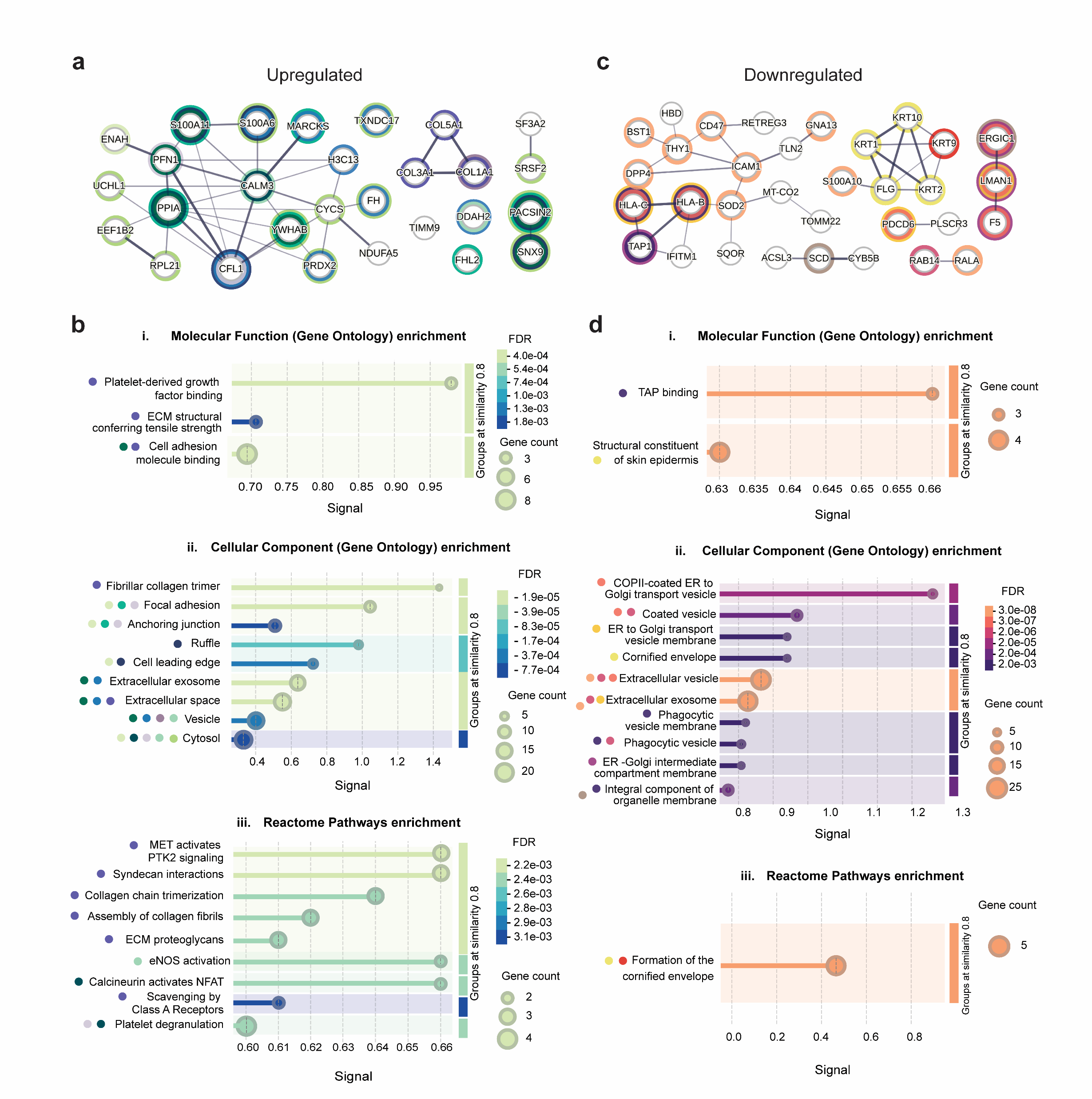


**Fig. S15 | Main biological changes induced by BMP-7 cmRNA transfections using the LipoMM lipid vector.** (**a**) Protein interaction networks generated from proteomic analysis of BMP-7 cmRNA-transfected hMSCs, with non-coding cmRNA-transfected hMSCs as controls. Line thickness represents the strength of data support, and color of circles around each node indicate enrichment categories. (**i**) Network for upregulated proteins. (**ii**) Network for downregulated proteins. (**b**, **c**) Graphs illustrating key biological changes, including: (**i**) molecular functions, (**ii**) cellular components, and (**iii**) Reactome pathways. The y-axis represents enriched terms, while the x-axis shows their signal (a metric combining the observed/expected ratio and -log(FDR)). Line colors represent the FDR scale, and circle size indicates gene count. Color of circles at the left of each term relate to protein network in (**a**). Protein-protein interactions and enrichments were conducted using the STRING tool.

**Fig. S16 | Metascape analysis of statistically enriched proteomic terms**. hMSCs were transfected with BMP-7 cmRNA delivered using (**a**) NL37 or (**b**) LipoMM vectors, and protein samples were collected two days post-transfection. Enriched terms were identified by comparing the results to non-coding cmRNA-transfected hMSC controls. Red arrows highlight key enriched terms relevant to bone regeneration pathways.


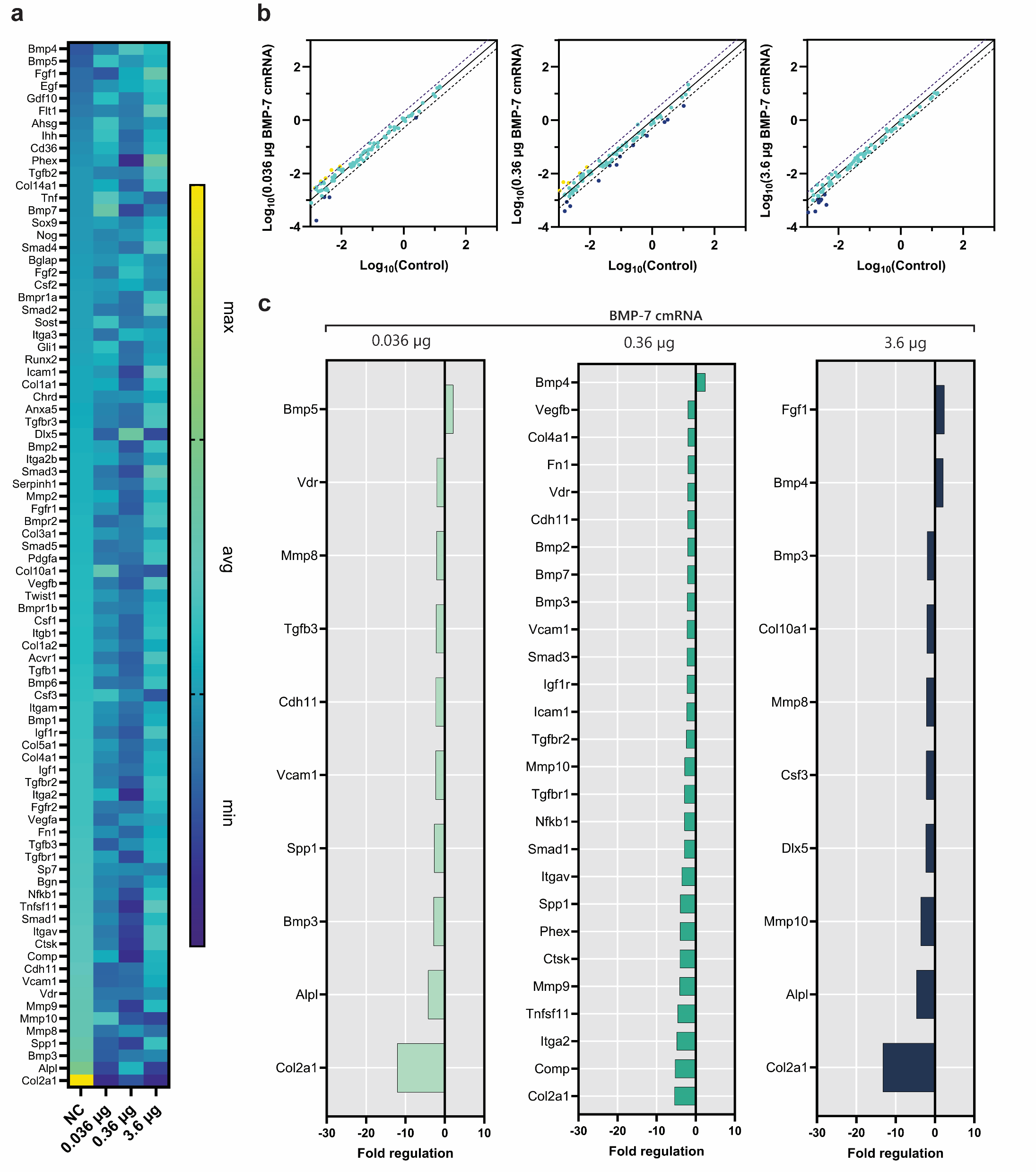


**Fig. S17 | Osteogenic gene expression in newly formed tissue after 2 weeks of implantation.** (**a**) Heat maps illustrating the magnitude of gene expression of all osteogenic markers. (**b**) Scatter plots comparing normalized gene expression between treatment groups and the NC control group. (**c**) Bar graphs showing fold changes in gene upregulation and downregulation, calculated using the 2^-ΔΔCT^ method.


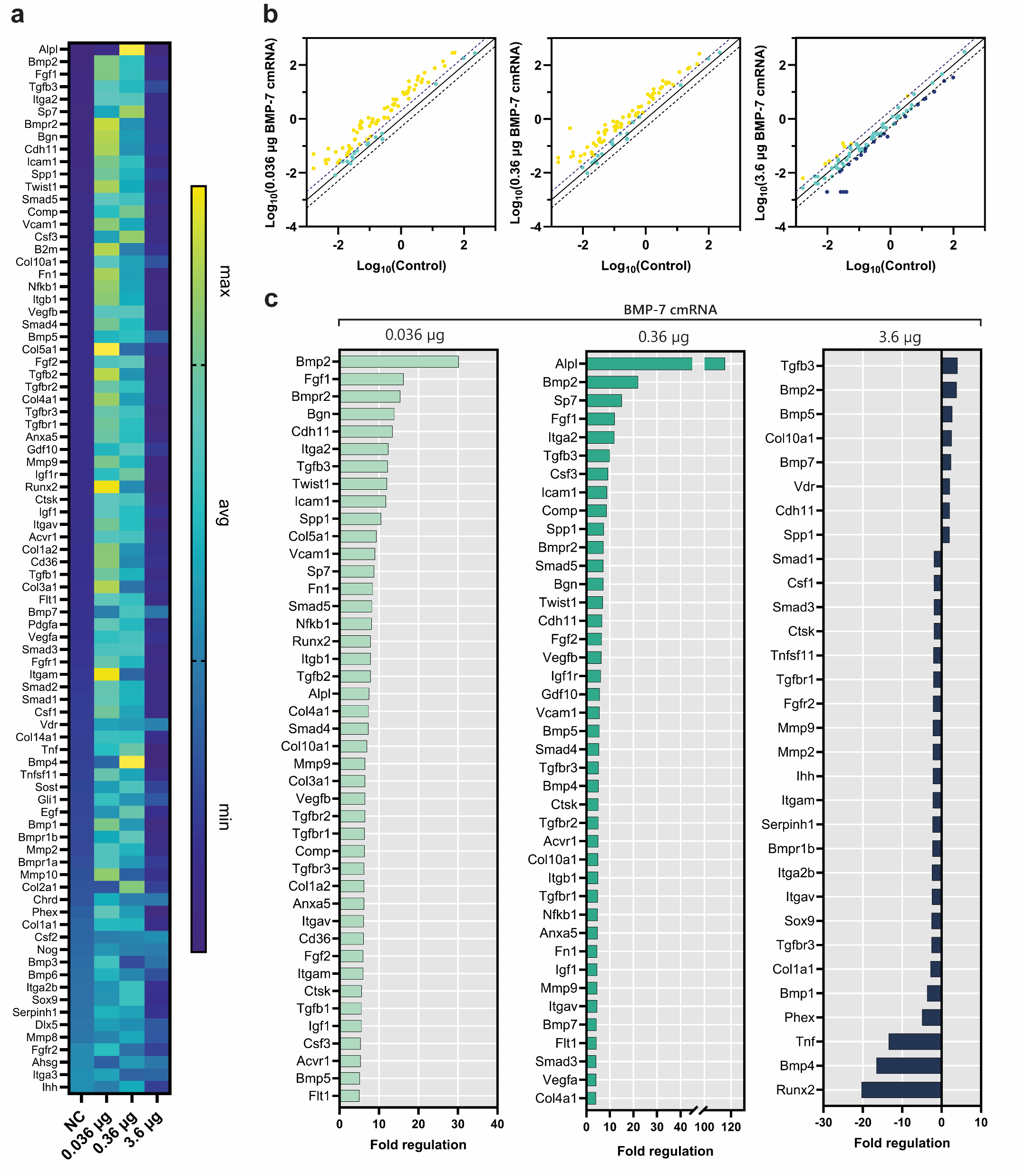


**Fig. S18 | Osteogenic gene expression in newly formed tissue after 4 weeks of implantation.** (**a**) Heat maps illustrating the magnitude of gene expression of all osteogenic markers. (**b**) Scatter plots comparing normalized gene expression between treatment groups and the NC control group. (**c**) Bar graphs showing fold changes in gene upregulation and downregulation, calculated using the 2^-ΔΔCT^ method.


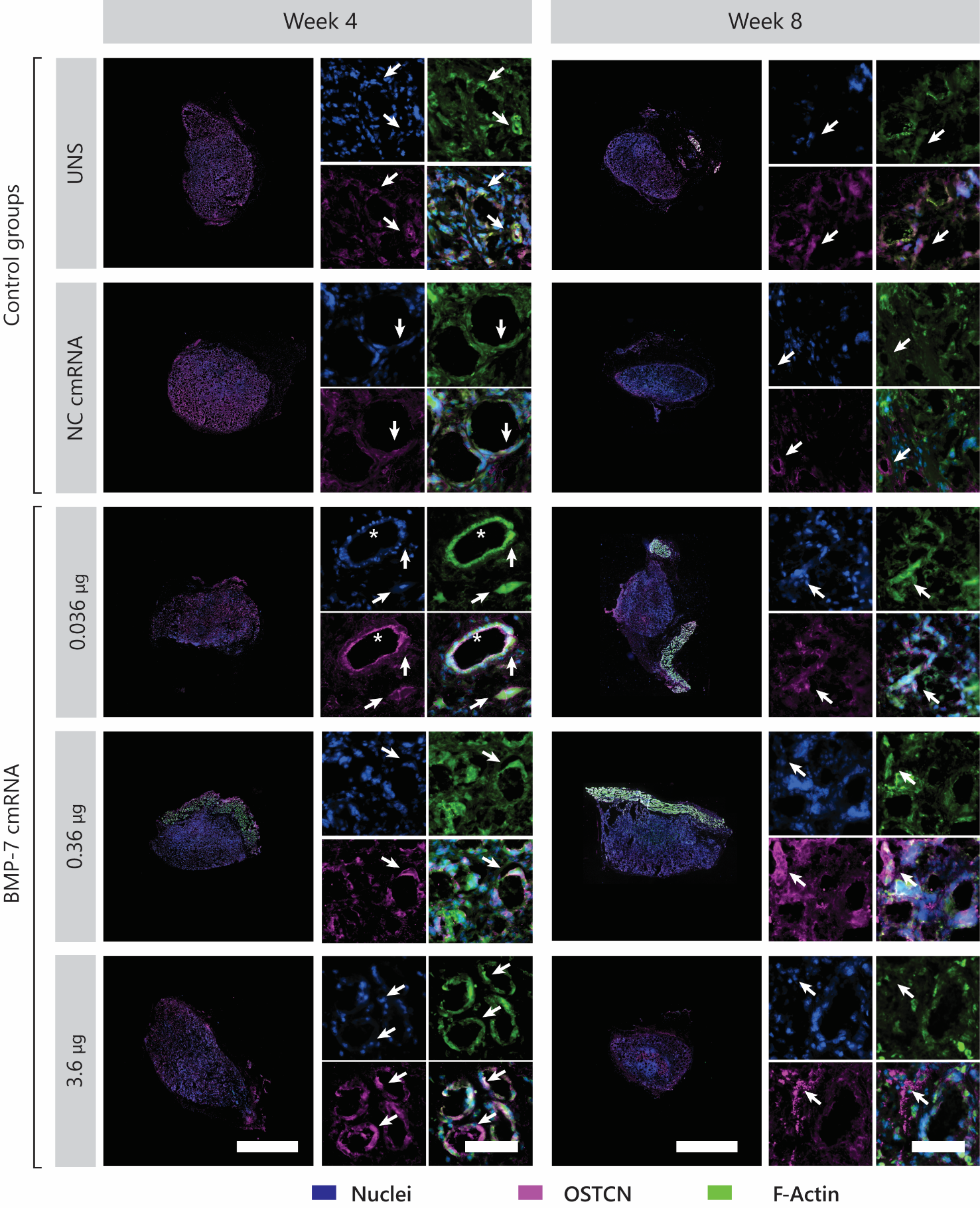


**Fig. S19 | Osteocalcin and F-actin Immunostainings of Explants.** Representative images of scaffolds collected at 4 and 8 weeks post-implantation. DAPI (blue) stains nuclei, osteocalcin (OSTCN, magenta) highlights osteoblast activity, and phalloidin (green) marks F-actin filaments. In each time point, the left images displays an overview of the entire scaffold area, while the right images presents magnified regions, with characteristic OSTCN and F-actin-positive osteoblasts indicated by white arrows. White asterisks show bone lining –like cells. Scale bars: 2 mm (overview) and 100 µm (magnified view).


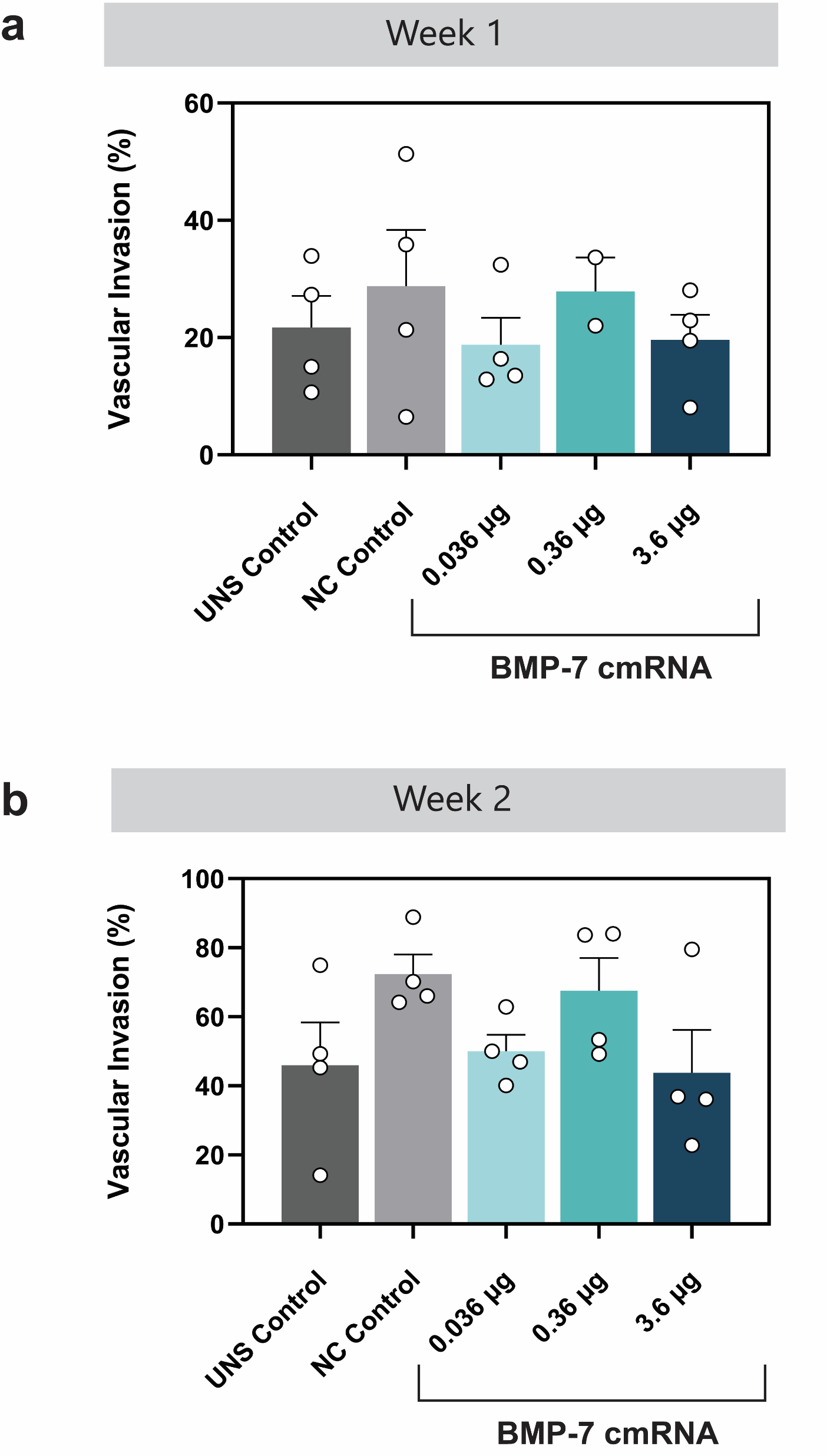


**Fig. S20 | Percentage of Vascular Invasion in Explants.** Quantification of vascular invasion after 1 (**a**) and 2 weeks post-implantation (**b**). Data are presented as mean ± SD (n=4 images/condition). A two-way ANOVA with Dunn’s multiple comparison test revealed no significant differences between groups.
